# Polyphenolics and Chemical Profiles of Domestic Norwegian Apple (*Malus* × *domestica* Borkh.) Cultivars

**DOI:** 10.3389/fnut.2022.941487

**Published:** 2022-06-30

**Authors:** Milica Fotirić Akšić, Milica Nešović, Ivanka Ćirić, Živoslav Tešić, Lato Pezo, Tomislav Tosti, Uroš Gašić, Biljana Dojčinović, Biljana Lončar, Mekjell Meland

**Affiliations:** ^1^Faculty of Agriculture, University of Belgrade, Belgrade, Serbia; ^2^Institute of General and Physical Chemistry, Belgrade, Serbia; ^3^Innovative Centre Faculty of Chemistry Belgrade, University of Belgrade, Belgrade, Serbia; ^4^Faculty of Chemistry, University of Belgrade, Belgrade, Serbia; ^5^Department of Plant Physiology, Institute for Biological Research “Siniša Stanković”, National Institute of Republic of Serbia, University of Belgrade, Belgrade, Serbia; ^6^Institute of Chemistry, Technology and Metallurgy, National Institute of the Republic of Serbia, University of Belgrade, Belgrade, Serbia; ^7^University of Novi Sad-Faculty of Technology Novi Sad, Novi Sad, Serbia; ^8^Department of Horticulture, NIBIO Ullensvang, Norwegian Institute of Bioeconomy Research, Lofthus, Norway

**Keywords:** antioxidant capacity, genetic resources, sugars, organic acids, polyphenols, minerals

## Abstract

Using modern analytical techniques, a comprehensive study of the chemical composition of fruits from apple cultivars grown in Western Norway during 2019 and 2020 was done. Metals, sugars, organic acids, antioxidant tests, and polyphenol content have been observed. In all investigated samples, the most dominant sugars were glucose, fructose, and sucrose. Among 11 tested organic acids, the dominant was malic acid, followed by citric and maleic acid. The most common metal was potassium, followed by magnesium and zinc. The quantification of polyphenols showed that among the 11 quantified polyphenols, chlorogenic acid, quercetin 3-*O-*rhamnoside, quercetin 3*-O*-glucoside, quercetin, and phlorizin were the most abundant. A detailed study of the polyphenolic profile of nine investigated apple samples provided 30 identified polyphenolic compounds from the class of hydroxybenzoic and hydroxycinnamic acids, flavonoids, and dihydrochalcones. In addition to the identified 3-*O*-caffeoylquinic acid, its two isomers of 5-*O*-caffeoylquinic acid and three esters were also found. Present polyphenols of the tested apples provided significant data on the quality of Norwegian apples, and they contribute to the distinguishing of these apple samples.

## Introduction

The ancestor of today's apple (*Malus* × *domestica* Borkh.) comes from the Tian Shan Mountains (Central Asia) and is thought to have originated about 4,000–10,000 years ago. Throughout history, apples were hybridized with other *Mauls* species and traveled along the Silk Road westward. Later, Greeks and Romans distributed apples throughout Europe (1). It is one of the most important temperate fruit species, and with 86 million tons it ranks the second place in the world's fruit production, while China (47% of world production) is the leading country (2). In Norway, apple production (on more than 1,500 ha and more than 12,000 tons) is organized mostly around fjords, which are the most northerly fruit tree-producing area in the world (3).

Apple and apple products (jam, juice, concentrate, marmalade, compotes, tea, wine, dried, cider) are rich in various products of primary and secondary metabolism which are also associated with general human health. Their quantities depend on genotype, rootstock, agro-technical measurements (fertilizing, irrigation, and pruning), growing season, picking time, storage, and processing together with biotic and biotic stresses (4). The quantity and ratio of sugars in the fruit depend on cultivar/genotype, ripening time, soil and microclimatic conditions, orchard maintenance, and all kinds of biotic and abiotic stresses (5). Apples are recognized as an excellent source of carbohydrates, where glucose, fructose, and sucrose are the most abundant (6, 7). Malic acid is the dominant organic acid in apple fruits, sometimes rich in up to 90% of the total organic acids, followed by citric, shikimic, fumaric, and quinic acid (8, 9). If acids are too low, the sweet taste becomes predominant and bland. The balance between sugars and acids has an important role in consumer acceptance where apple cultivars with sugar/acid ratios lower than 20 are sharp and appropriate for processing and cider production, while others can be used for table consumption (10).

Roussos and Gasparatos (11) and Fotirić Akšić et al. (12) found that apple fruits are a good source of minerals, especially K, P, Ca, Mg, N, Zn, Fe, and others. Minerals are involved in many physiological processes within the apple fruit. Calcium, potassium, and magnesium and their ratios in mature apple fruits influence their quality, cell wall structure, and storage life, while its deficiency can provoke physiological disorders (13, 14). Nitrogen is one of the most important minerals for the fruit tree physiology, and if too little it will inhibit growth and fruit set but if too much it will induce problems with fruit quality, excessive vegetative growth, and diseases (15). In contrast, minerals are involved in the metabolism of carbohydrates, lipids, proteins, vitamins, and enzymes in humans and their consumption can lower lung dysfunctions and various cancers, particularly prostate, liver, colon, and lung (16).

One of the largest groups of secondary plant metabolites in apple fruit are polyphenols that account for the fruit color, flavor, and taste of the fruit (17). The apple peel has several times higher phenolic content than apple pulp that can be partly transferred to the corresponding juice (7, 18, 19). The concentration of polyphenols is influenced by the genotype, all kinds of biotic and abiotic stresses, environmental factors (season, different latitudes, soil, light exposure), as well as agricultural techniques (conventional/integrated/organic cultivation, fertilization, irrigation) (20). The most common phenolic compounds that could be quantified in apple fruits are flavan-3-ols (catechin, epicatechin), phenolic acids (chlorogenic acid, caffeic acid, ferulic acid, protocatecuic acid), flavonoids (rutin, baicalein, and naringenin), flavonols (quercetin glycosides, kaempherol), dihydrochalcones (phloridzin, phloretin), and anthocyanins (cyanidin-3-*O*-galactoside) (7, 21). Additionally, phenolic compounds, especially proanthocyanidins and phlorizin, contribute to the astringency and bitterness of apples, while chlorogenic acid is a non-bitter phenolic acid (22). The presence of specific phenolic compounds can cause resistance of apple cultivar to the most important diseases. In such a way, phloridzin (phytoalexin) provides resistance to plant pathogens such as apple scab and bacterial cancer (23). Apples have high antioxidant activity and prevent chronic diseases such as cancers, asthma, aging, and cognitive processes, Alzheimer's disease, and improve bone and gastrointestinal health and pulmonary function (17). Consumption of apples lowers the level of cholesterol and triglycerides in the blood and reduces cardiovascular diseases, obesity, and diabetes (24).

Due to the accumulation of the somatic mutations, spontaneous hybridization, selection, and human activities during the long history of its cultivation, the genus *Malus*, at present, is characterized by a large diversity (25). Even with more than 10,000 cultivars recognized, only 10 of those (Idared, McIntosh, Cox's Orange Pippin, Cripps Pink, Honey Crisp, Braeburn, Fuji, Gala, Granny Smith, Red Delicious, and Golden Delicious) are grown worldwide (26). However, great quantities of apples are produced in small-scale orchards, established with local, stress-resistant cultivars, having good morphological and pomological attributes that can be even superior to “top 10” apple cultivars and form a huge reservoir of variability (27). Recently, the nostalgia for old varieties has increased since a certain degree of a monotony of taste is offered in the mainstream supermarkets. Besides, archive apple cultivars (apple germplasm) are an important pool of genetic diversity, which are a carrier of genes that influence the ability of the genotype to adapt to the changing environments, pests, and improve quality *via* hybridization in some breeding programs.

For these reasons, the aim of this study was to analyze the chemical content of 103 apple archive cultivars that are grown in Norway and carefully select nine samples for further analysis of phenolic profile. This kind of study will provide important information about the apple samples due to the well-known amplification of chemical compounds as markers of growing location.

## Materials and Methods

### Chemicals and Standards

Sugar standards (trehalose, arabinose, glucose, fructose, sucrose, turanose, galactose, ribose, isomaltose, isomaltotriose, maltose, maltotriose, xylose, melibiose, panose, rhamnose, raffinose, stachyose) were purchased from Tokyo Chemical Industry (TCI, Zwijndrecht, Belgium); standards of sugar alcohols (sorbitol, glycerol, galactitol, mannitol), organic acids standards (citric, maleic, malic, pyruvic, shikimic, lactic, propionic, butyric, quinic, oxalic, fumaric acids), sodium acetate trihydrate, sodium hydroxide, methanol, phenolic standards (3-*O*-caffeoylquinic acid, caffeic acid, chlorogenic acid, ferulic acid, gallic acid, *p*-coumaric acid, *p*-hydroxybenzoic acid, *p*-hydroxyphenyl acetic acid, protocatechuic acid, sinapic acid, syringic acid, vanillic acid, acacetin, aesculetin, catechin, eriodictyol, isorhamnetin 3-*O*-glucoside, isorhamnetin 3-*O*-rutinoside, kaempferol, kaempferol 3-*O*-glucoside, kaempferol 7-*O*-glucoside, naringenin, naringin, phloretin, phlorizin, quercetin, quercetin 3-*O*-glucoside, quercetin 3-*O*-rhamnoside, rutin), Trolox standard, and gallic acid were from Sigma-Aldrich (Steinheim, Germany). Formic acid, acetonitrile, nitric acid, and hydrogen peroxide were from Merck (Darmstadt, Germany); multi-element plasma standard solution 4 was from Alfa Aesar GmbH & Co KG (Kandel, Germany) and ILM 05.2 ICS Stock 1 was from VHG Labs, Inc, part of LGC Standards (Manchester, USA). Strata C18-E type (500 mg/3 ml) cartridges for solid-phase extraction (SPE) were purchased from Phenomenex (Torrance, CA) and syringe filters (15 mm; 0.45 μm and 0.22 μm) were from Supelco (Bellefonte, PA). All aqueous solutions were prepared using ultrapure water (0.055 mS/cm) obtained by using the Thermo Fisher TKA MicroPure water purification system.

### Plant Material

Apple fruits (Supplementary Table S1) were collected from two areas in Western Norway, the experimental farm of NIBIO Ullensvang (latitude 60°19′8.03″N, longitude 6°39′14.31″E) and Njøs Fruit and Berry Center, Leikanger (at latitude 61°10′43.2″N, longitude 6°51′34.3″E), along the Sognefjord. All apple trees were trained as spindle trees and pruned to a maximum height of about 2.5–3 m. In both orchards, the selected trees were homogeneous in terms of amounts of flowers vigor and health status. Orchard floor management consisted of grass in the interrows and a 1-m wide herbicide strip in the intrarow space, which is the industry standard for managements. The soil was a sandy-loam with ~5% organic matter. The trees were irrigated by drip irrigation when water deficits occurred. Based on soil and leaf analysis, all trees received the same amount of fertilizers. Fertilization and crop protection were carried out according to standard local fruit-growing practices. All trees received the same amount of fertilizers based on soil analysis. Hand thinning was carried out at both locations at the end of June to achieve optimum crop loads of good fruit quality (15 cm apart between fruitlets).

**Figure 1:**
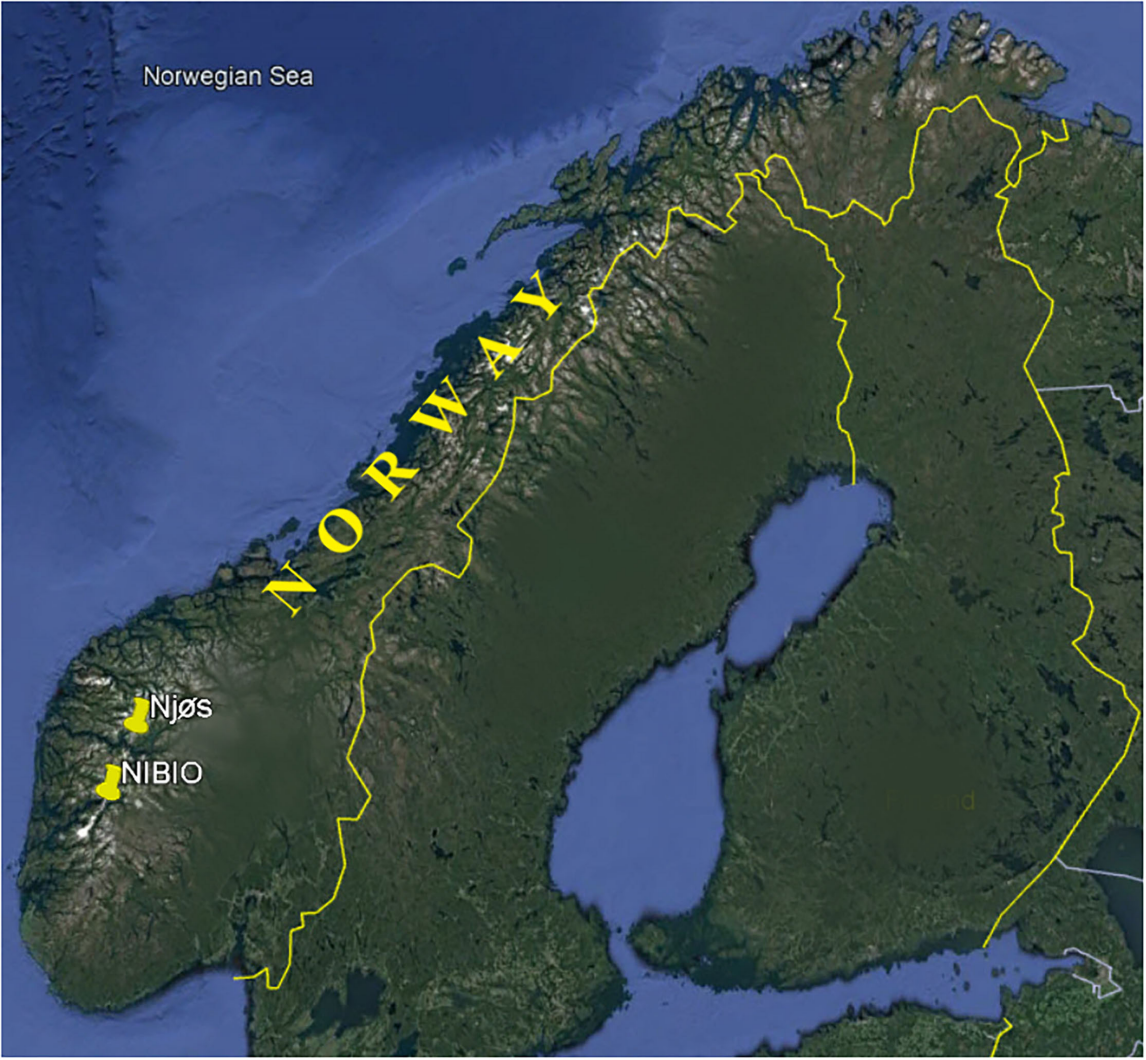
Sampling sites of apple fruits in Norway. NIBIO, Norwegian Institute of Bioeconomy Research, Ullensvang area, and Njøs.

Fruits from 74 apple cultivars, at optimal harvest times based on parameters such as ground color, firmness, taste, and seeds color, were picked from the Norwegian Institute of Bioeconomy Research (NIBIO) Ullensvang area and fruits from 29 apple cultivars from Njøs Fruit and Berry Center (Figure 1, Supplementary Table S1). Among those 103 apple samples, four apple varieties “Franskar,” “Fuhr,” “Furuholm,” and “Løeple” were grown in both areas. The apple samples were collected during the 2-year seasons, 2019 and 2020. The fruits were cut into small pieces and dried in an oven at 40°C for about 10 days. All samples were grounded to powder using an analytical mill (A 10 basic Analytical mill, IKA-Werke GmbH & Co). The samples were measured in duplicate and used for further analysis. All results are expressed on the dry weight (dw) of the sample.

### Weather Conditions

West Norway, especially the fjord areas, has a cool, maritime, Nordic climate, which is under the influence of the Gulf Stream. It is characterized by cool summers and mild winters. Weather fronts are usually coming from southwest from the North Sea and the Atlantic Ocean. It rarely has problems with frost damage of the fruit trees, either during the winter or during blossom time. The snow-covered mountains give protection to high amounts of rain from the west. In Ullensvang, the average temperature for the year is 7.6°C and the annual rainfall 1,705 mm. May, June, and July are the driest months (78, 75, and 77 mm, respectively) and December is the coldest and has the highest rainfall (1.7°C and 243 mm rain in average). The average temperature during the season, May–September, is 13.4°C. The average annual air temperature at Njøs is 7.3°C (during spring 4–11°C), annual rainfall is 1,063 mm (with May being driest with 15 rainy days) and an average annual humidity percentage of 78.0%. Average monthly hours of sunshine are the highest in May (~175 h), and the mean monthly wind speed over the year is 3 m/s.

### Preparation of Extracts

For sugar and organic acid determination, 1.0 g of homogenized sample was 100-fold diluted with ultrapure water and sonicated for 30 min. The solutions were centrifuged at 9,000 rpm/min for 20 min and supernatants were filtered through a 0.22 μm syringe filter. The filtrate was kept at −18°C until analysis.

In the case of phenolic analysis and antioxidant activity, apple extract samples were prepared following the procedure described by Pavlović et al. (28) with slight modifications. To the measured amount of 0.4–0.5 g of each dry apple sample, an amount of 10 ml of acidified methanol/water solution (70/30 with 0.1% hydrochloric acid to pH 2) was added. After using the ultrasonic bath (twice for 30 min), the samples were centrifuged (6,000 rpm/min for 10 min) and passed through 0.45 μm syringe filters. For further chromatographic analysis, SPE was used to isolate and concentrate polyphenolic compounds in sample extracts.

### Determination of Metals by ICP-MS

#### Microwave Digestion

The digestion of finely chopped and homogenized samples was performed on the Advanced Microwave Digestion System (ETHOS 1, Milestone, Italy) using an HPR-1000/10S high-pressure segmented rotor. About 0.25 g of sample were mixed with 10 ml HNO_3_ (65 wt.%) and 1 ml H_2_O_2_ (30 wt.%). The temperature was gradually raised with microwave power (0–1,000 W) to 200°C in the first 20 min, remained at 200°C in the next 20 min, and then decreased rapidly to room temperature. After cooling and without filtration, the solution was diluted to a fixed volume (25 ml) with ultrapure water.

#### ICP-OES Measurement

The content of major and trace elements in solution samples was determined by inductively coupled plasma optic emission spectrometry (ICP-OES). ICP-OES measurement was performed using Thermo Scientific iCAP 6500 Duo ICP (Thermo Fisher Scientific, Cambridge, UK) spectrometer equipped with RACID86 Charge Injector Device (CID) detector. The optical system was purged with argon and the Echelle polychromator was thermostated at 38°C. The instrumental operating conditions for ICP-OES are shown in Supplementary Table S2.

The calibration blank was repaired by acidifying reagent water to the same concentrations of the acids found in the standards and samples. The method blank must contain all of the reagents in the same volumes as used in the processing of the samples. The method blank must be carried out through the complete procedure and contain the same acid concentration in the final solution as the sample solution used for analysis. Concentrations of elements of the sample are expressed as mg/kg.

### Determination of Sugars and Sugar Alcohols by IC

A high-performance anion-exchange liquid chromatography system with pulse amperometric detection was used to analyze sugar and sugar alcohols. Chromatographic measurement was performed using DIONEX ICS 3000 DP liquid chromatography system (Dionex, Sunnyvale, CA, USA) equipped with a quaternary gradient pump and electrochemical detector, which consisted of Au as the working electrode and Ag/AgCl as reference electrode. All separations were performed on Carbo Pac®PA100 (4 × 250 mm; Dionex, Sunnyvale, CA, USA) thermostated to 30°C. The flow was constant (0.7 ml/min), whereas the composition of the mobile phase is given in Supplementary Table S3.

### Determination of Organic Acids by IC

The organic acid analysis was performed on Dionex ICS 3000 associated with a single-channel pump, conductivity detector (ASRS ULTRA II [4 mm], recycle mode), eluent generator (KOH), and Chromeleon software (Chromatography Workstation and Chromeleon 6.7 Chromatography Management Software). All separations were made on the analytical column IonPac AS15, 4 × 250 mm and IonPac AG15 guard column, 4 × 50 mm. The mobile phase flow rate was 0.5 ml/min, and mobile phase composition was changed (gradient elution) during the analysis in the following order: 0–4 min = 10 mM KOH; 4–20 min = from 10 mM to 60 mM KOH; 20–30 min = from 60 mM to 10 mM; 30–35 min = 10 mM.

### Polyphenolic Analysis

A mixture of each phenolic standard was prepared in methanol as a 1,000 mg/L stock solution. Dilution of the stock solution with methanol yielded working solutions at concentrations of 0.025, 0.050, 0.100, 0.250, 0.500, 0.750, and 1.000 mg/L. They were kept in the dark at 4°C. Calibration curves were obtained by plotting the peak areas of the standards against their concentration.

Polyphenolic were quantified using an ultra-high-performance liquid chromatography system (UHPLC) coupled with diode array detector (DAD) and mass spectrometer. The polyphenolic compounds were separated using a Dionex Ultimate 3000 UHPLC system. Separation was achieved on a Syncronis C18 analytical column (100 × 2.1 mm i.d., 1.7 μm, Thermo Fisher Scientific, Bremen, Germany). The mobile phase consisted of (A) water + 0.1% formic acid and (B) acetonitrile. The mass spectrometer TSQ Quantum Access Max triple quadruple (QqQ) was operated in negative mode from *m/z* 100 to 1,000. To quantify the polyphenols, a molecular ion and two of the most intense fragments from the MS^2^ spectrum were recorded in particular. Detailed chromatographic and MS parameters were previously published in Gašić et al. (29). The Xcalibur software (version 2.2) was used for instrument control. Polyphenols were quantitated according to the corresponding spectrometric characteristics of reference standards. Total contents of all compounds were obtained by plotting the peak areas and expressed as mg/L.

In addition, phenolic profiles of nine chosen apple samples were analyzed on a UHPLC system connected to a linear trap quadrupole (LTQ) OrbiTrap mass spectrometer (Thermo Fisher Scientific, Bremen, Germany). The following conditions of both chromatography and spectrometry parts of UHPLC-LTQ OrbiTrap MS were described in our previously study (29). The MS^2^, MS^3^, and MS^4^ fragmentations were confirmed using available standards or published fragmentation data.

### Data Evaluation for Correlation Study

Principal component analysis (PCA) facilitated understanding of patterns in analyzed data by offering information on determining variables, which behave similarly to each other. The results of PCA of the 103 samples (nos. 1–74 from the NIBIO Ullensvang area, and nos. 75–103 from Njøs) according to investigated variables such as the content of nutrients, sugars, organic acids, and polyphenolics, were represented in biplots. Data were examined using StatSoft Statistica 12 (StatSoft Inc., Tulsa, OK, USA).

## Results and Discussion

### Chemical Analysis

The chemical analysis of fruits from the 103 investigated apple cultivars included results for sugar compounds, organic acids, minerals, phenolic compounds, and antioxidant tests [total phenolic content (TPC) and relative scavenging activity (RSA)]. All results were presented by descriptive analysis, with minimum, maximum, mean value, and standard deviation, which were done on average values over 2 years, for apple samples from the NIBIO Ullensvang area, and Njøs (Tables 1–4).

**Table 1:** Descriptive analysis of sugar content (g/kg dw) in Norwegian apple samples from the NIBIO Ullensvang area (no. 1–74) and from Njøs (no. 75–103) during 2-year harvesting.

**Norwegian area**	**NIBIO Ullensvang area**				**Njøs**			
**Parameter**	**Min**	**Max**	**Mean**	**SD**	**Min**	**Max**	**Mean**	**SD**
**Sorbitol**	9.53	82.53	42.81	17.22	7.56	61.17	20.21	13.39
**Trehalose**	0.29	41.40	9.55	9.39	0.31	22.96	3.15	5.02
**Arabinose**	0.26	18.05	4.50	4.99	0.14	11.34	1.67	2.52
**Glucose**	153.10	256.93	201.16	23.45	137.76	225.60	180.18	17.88
**Fructose**	208.77	313.47	260.42	27.23	161.82	240.86	200.31	18.71
**Sucrose**	83.49	297.35	193.14	36.25	126.90	210.19	148.75	17.32
**Turanose**	1.68	38.64	20.06	6.82	1.70	36.10	15.03	9.42
**Glycerol**	1.66	32.75	8.57	6.68	0.97	44.22	4.36	8.44
**Galactitol**	1.21	12.22	4.89	2.83	1.22	7.99	2.81	1.46
**Galactose**	0.34	25.63	7.25	4.46	0.87	11.98	4.15	3.13
**Ribose**	1.99	34.06	11.39	7.31	1.69	21.65	6.60	4.85
**Isomaltose**	1.32	12.33	5.68	2.82	0.78	13.01	2.87	3.03
**Isomaltotriose**	0.51	5.71	2.08	1.27	0.52	5.15	1.12	0.88
**Maltose**	0.48	10.99	3.97	2.79	0.37	7.05	1.94	1.48
**Maltotriose**	0.84	5.46	1.97	1.13	0.59	3.56	1.23	0.69
**Mannitol**	0.12	15.07	4.05	3.86	0.11	8.78	2.64	1.85
**Xylose**	0.30	17.20	6.10	4.09	0.38	11.61	4.97	2.64
**Melibiose**	0.30	18.07	6.53	4.08	0.52	11.84	2.57	2.89
**Panose**	0.22	7.83	2.45	1.81	0.24	5.14	0.82	1.08
**Rhamnose**	0.26	12.45	2.87	2.51	0.73	5.89	2.44	1.39
**Raffinose**	0.61	16.25	3.84	2.74	1.28	10.56	3.52	3.19
**Stachyose**	0.47	4.86	1.53	0.95	0.51	3.45	1.08	0.65
**Sum of sugars**	593.01	848.06	744.54	54.86	522.08	792.56	582.42	56.07
**Sum of sugar alcohols**	20.55	105.18	60.33	21.16	14.03	110.63	30.01	20.57

**Table 2:** Descriptive analysis of organic acids content (g/kg dw) in Norwegian apple samples from the NIBIO Ullensvang area (no. 1–74) and from Njøs (no. 75–103) during 2-year harvesting.

**Norwegian area**	**NIBIO Ullensvang area**				**Njøs**			
**Parameter**	**Min**	**Max**	**Mean**	**SD**	**Min**	**Max**	**Mean**	**SD**
**Citric**	6.89	30.17	14.77	5.96	1.70	16.19	3.34	2.83
**Maleic**	4.74	56.23	16.55	10.77	2.22	10.80	4.77	2.09
**Malic**	15.72	102.30	45.68	19.14	6.89	46.08	17.10	8.58
**Pyruvic**	0.11	2.54	1.22	0.49	0.71	2.02	1.19	0.33
**Shikimic**	0.02	1.57	0.36	0.26	0.02	1.59	0.17	0.36
**Lactic**	0.42	2.68	1.01	0.43	0.74	2.34	1.48	0.35
**Propionic**	0.18	4.50	1.44	0.92	0.55	3.09	1.29	0.57
**Butiric**	0.52	2.85	1.15	0.57	0.06	2.03	0.43	0.60
**Quinic**	0.84	24.26	3.70	3.71	0.32	5.89	1.42	1.24
**Oxalic**	0.91	10.18	3.59	2.24	0.32	8.21	2.57	1.86
**Fumaric**	0.64	2.49	1.37	0.46	0.95	3.96	2.03	0.80

**Table 3:** Descriptive analysis of content of nutrients (mg/kg dw) in Norwegian apple samples from the NIBIO Ullensvang area (no. 1–74) and from Njøs (no. 75–103) during 2-year harvesting.

**Norwegian area**	**NIBIO Ullensvang area**				**Njøs**			
**Parameter**	**Min**	**Max**	**Mean**	**SD**	**Min**	**Max**	**Mean**	**SD**
**Al**	5.65	210.75	51.97	42.41	3.23	44.03	13.16	11.45
**B**	6.95	54.89	19.01	12.47	5.17	65.10	18.18	16.28
**Ca**	13.97	901.19	325.65	125.65	25.45	313.13	112.91	87.96
**Cu**	1.12	27.67	2.94	3.01	0.76	2.99	1.72	0.47
**Fe**	1.81	644.59	40.76	82.80	2.20	27.62	8.20	5.61
**K**	1,377.23	7,570.56	5,239.41	1,208.13	1,379.70	3,946.52	2,451.02	776.82
**Mg**	84.45	388.87	266.64	66.37	38.52	212.49	103.85	50.50
**Mn**	0.37	6.97	2.28	0.94	0.30	2.24	0.97	0.55
**Na**	14.10	582.85	129.98	92.03	0.27	157.64	54.65	38.46
**Zn**	3.30	12,626.49	4,499.88	1,956.57	5,288.89	23,888.72	9,805.33	4,458.70
**P**	112.57	673.91	375.32	88.99	149.98	449.02	270.87	74.60
**S**	1.81	489.46	156.82	70.01	2.87	81.07	8.02	14.48
**N (%)**	4.03	6.22	4.83	0.45	3.74	6.23	4.82	0.61

**Table 4:** Descriptive analysis of the content of phenolic compounds (mg/kg dw), total phenolic content (TPC, g GAE/kg dw), and relative scavenging activity (RSA, mmol TE/kg dw) in Norwegian apple samples from the NIBIO Ullensvang area (no. 1–74) and from Njøs (no. 75–103) during 2-year harvesting.

**Norwegian area**	**NIBIO Ullensvang area**				**Njøs**			
**Parameter**	**Min**	**Max**	**Mean**	**SD**	**Min**	**Max**	**Mean**	**SD**
**3-** ***O*** **-Caffeoylquinic acid**	0	579.58	303.85	128.58	0	138.22	47.95	25.20
**Caffeic acid**	0.31	5.99	2.15	1.17	0	8.12	1.84	2.28
**Chlorogenic acid**	28.40	904.72	323.97	200.36	0	664.08	174.87	244.74
**Ferulic acid**	0.15	3.03	1.19	0.66	0	1.84	0.70	0.43
**Gallic acid**	0.02	14.53	1.16	2.83	0	0.09	<0.01	0.02
***p*** **-Coumaric acid**	0.37	7.56	1.94	1.38	0	3.69	0.71	0.94
***p*** **-Hydroxybenzoic acid**	0.17	10.16	1.42	1.33	0.07	2.34	0.33	0.44
***p*** **-Hydroxyphenylacetic acid**	0	0.89	0.09	0.14	0	0	0	0
**Protocatechuic acid**	0.86	20.36	5.36	4.70	0	9.97	1.18	2.51
**Sinapic acid**	0	0.57	0.04	0.11	0	38.50	1.33	7.15
**Syringic acid**	0	16.48	0.60	2.19	0	5.86	0.36	1.14
**Vanillic acid**	0.15	0.85	0.36	0.01	0	0.42	0.01	0.08
**Acacetin**	0	1.41	0.29	0.29	0	0.38	0.13	0.11
**Aesculetin**	0	0	0	0	0	9.15	1.84	2.65
**Catechin**	0	98.98	8.63	19.91	0	0	0	0
**Eriodictyol**	0	0.54	0.10	0.09	0	0.07	<0.01	0.01
**Isorhamnetin 3-** ***O*** **-glucoside**	0.06	26.65	5.25	4.42	0	19.89	7.32	3.79
**Isorhamnetin 3-** ***O*** **-rutinoside**	0.10	6.06	1.30	1.26	0.08	6.61	0.89	1.50
**Kaempferol**	0	0.81	0.24	0.23	0	1.19	0.12	0.27
**Kaempferol 3-** ***O*** **-glucoside**	0	0	0	0	0	23.64	8.08	6.25
**Kaempferol 7-** ***O*** **-glucoside**	1.00	8.44	2.90	1.52	0	39.57	16.11	8.00
**Naringenin**	0	0.34	0.12	0.07	0	0.17	0.01	0.03
**Naringin**	0	13.33	3.37	2.82	0	7.20	2.29	1.78
**Phloretin**	0	2.27	0.36	0.29	0	6.41	0.79	1.19
**Phlorizin**	5.97	118.09	55.47	30.70	0	581.13	65.14	102.70
**Quercetin**	1.80	36.10	11.62	6.97	0	32.34	11.29	8.17
**Quercetin 3-** ***O*** **-glucoside**	3.67	86.47	33.01	15.66	0	56.56	22.68	13.25
**Quercetin 3-** ***O*** **-rhamnoside**	6.15	64.02	31.05	14.09	0	71.60	28.40	19.50
**Rutin**	0.07	30.88	6.27	6.65	0	28.78	6.12	7.44
**Sum of phenolic compounds**	177.45	1,684.72	802.13	324.37	53.89	953.43	400.5	290.26
**TPC**	3.47	20.59	8.43	3.13	5.86	17.34	9.00	2.49
**RSA**	5.63	103.52	39.18	17.64	33.69	128.24	66.57	21.20

#### Determination of Sugars

Sugars are primary products of photosynthesis, providing energy for all kinds of metabolic processes. Besides, sugars are important for fruit sweetness at harvest and consumers acceptance. In all investigated apple samples, 18 sugars and 4 sugar alcohols (Table 1) were determined. The most dominant sugars in apple samples were fructose, glucose, and sucrose. In the woody *Rosaceae* family, sucrose, glucose, and fructose and the sugar alcohol sorbitol are the most common (30). In addition, during the fruit ripening, sucrose is hydrolyzed into glucose and fructose. Following simple sugars, sorbitol and glycerol were the most abundant in studied apples. Sorbitol, as a very important translocated sugar, ranged from 7.56 to 82.53 g/kg dw. Our results correspond to those obtained by other studies (9, 31). Studied apples stored the lowest quantity of arabinose, panose, and mannitol (Table 1), whose minimum values were below 0.26 g/kg dw. The sugar accumulation was cultivar dependent, but external factors, such as weather conditions, type of soil, fertilization, and other treatments, influenced this process as well (32).

All sugar compounds showed higher mean values in apple samples from the Ullensvang area. However, two sugar compounds (glycerol and isomaltose) were found in higher content in apples from Njøs. The sum of the sugars was in the range from 522.08 to 848.06 g/kg dw, with higher mean values in the apple samples from the NIBIO Ullensvang area (Table 1). Furthermore, the sum of the sugar alcohols was two-fold higher in these apples (60.33 g/kg dw) in contrast to apples from Njøs (30.01 g/kg dw; Table 1).

#### Determination of Organic Acids

In fruits, organic acids are usually inversely related to sugar levels and strongly affect organoleptic quality like taste, sight, and smell. During maturation, organic acids accumulated in young fruits strongly decrease (33). The metabolism and accumulation of organic acids in fruits are under both genetic and environmental control (34). Organic acids play important functions in carbon to nitrogen and hormone metabolism, and in the control of fruit growth *via* cell expansion through water uptake (35).

In the studied apple fruits, malic acid was the most abundant organic acid, followed by maleic and citric acid. The results obtained in this study are in accordance with results from other authors (8, 9, 36). Samples from the NIBIO Ullensvang area had higher contents of organic acids compared to samples from Njøs. In the NIBIO Ullensvang samples, malic acid was the most dominant followed by maleic and citric acids with 2-year mean values of 45.68, 16.55, and 14.77 g/kg dw, respectively. The same trend (malic, maleic, citric acid) was observed in samples from Njøs with 2-year mean values of 17.10, 4.77, and 3.34 g/kg dw, respectively. The following organic acids in prevalence in the NIBIO Ullensvang samples were quinic and oxalic (2-year mean values 3.70 and 3.59 g/kg dw, respectively), while in the Njøs samples those were oxalic and fumaric acids (2-year mean values 2.57 and 2.03 g/kg dw, respectively). In literature (37), the existence of quinic acid in apple juice with a somewhat higher concentration was reported. Based on the obtained results, it can be concluded that Norwegian apples are, in general, rather acidic, which goes in line with other authors (38). According to Ma et al. (39), fruit acidity possibly underwent artificial selection during apple domestication, which means that autochthonous apple cultivars could be valuable resources for apple quality improvement.

#### Determination of Metals

Mineral content in apple fruits depends on stresses, biotic, and abiotic factors, as well as cultural practice (irrigation, rootstocks, and fertigation and foliar application of nutritional sprays). Apple minerals (especially N, K, P, Ca, and B) are very often correlated with fruit quality and disorders. Potassium, in most cases, increases fruit size, yield, acidity, and color, but decreases fruit firmness. Ca increases fruit firmness and lowers disorders (40).

Mineral contents in the investigated apple samples are presented in Table 3. In samples from the NIBIO Ullensvang area, potassium was the most dominant mineral with the 2-year mean of 5,239.41 ± 1,208.13 mg/kg, followed by zinc with a 2-year mean of 4,499.88 ± 1,956.57 mg/kg. In analyzed samples from Njøs, the order was reversed: the most abundant mineral was zinc (2-year mean 9,805.33 ± 4,458.70 mg/kg) followed by potassium (2-year mean 2,451.02 ± 776.82 mg/kg).

#### Determination of Polyphenolic Compounds and Polyphenolic Profile

The results of the UHPLC-DAD MS/MS showed the presence of 29 phenolic compounds, of which 12 were phenolic acids. The results were presented by descriptive analysis, which was done on average values over 2 years, for apple samples from the NIBIO Ullensvang area, and Njøs (Table 4). The dominant content of chlorogenic acid, 3-*O*-caffeoylquinic acid, and phlorizin was noted. These compounds contributed the most to the total sum of all polyphenolic compounds (Table 4). The next were quercetin 3-*O*-glucoside and quercetin 3-*O*-rhamnoside. Obtained values for chlorogenic acid, phlorizin, and quercetin derivatives were similar to those published for apples from various European countries (41, 42). The importance of the quercetin derivatives was observed for apple pomace by other authors (43), as well as the dominant content of 5-*O*-caffeoylquinic acid and phloretin derivatives (36).

It could be noted that many polyphenolic compounds showed higher content in apples from the NIBIO Ullensvang area, which provide a twice-higher sum of polyphenolic compounds (802.13 in contrast to 400.50 mg/kg dw for Njøs Table 4). Several polyphenolic compounds showed notable differences between apples from the two areas. Thus, aesculetin and kaempferol 3-*O*-glucoside did not quantify in the NIBIO apples, and *p*-hydroxyphenylacetic acid and catechin were absent in all samples from Njøs (Table 4). Moreover, gallic acid, vanillic acid, eriodictyol, and naringenin were also absent in most samples from Njøs, with the exception of one sample (no. 81, “Enestaende”), which explains the values of mean value and standard deviation in Table 4. Similar to that, sinapic acid was present in an amount of 38.50 mg/kg dw in one sample (no. 83, “Franskar” from Njøs) without it appearing in other Njøs samples, while its accumulation in NIBIO samples was diverse, and with lower content (up to 16.48 mg/kg dw). These results were in line with the statement of other authors about the different properties of apples that depend on the growing location (41). The results of phenolic compounds were similar to the values published by other authors (44), who analyzed peel and pulp in four different apple varieties. In addition to their results for apple pulp samples (44), the content of phenolic acids (such as chlorogenic acid, *p*-hydroxybenzoic acid, syringic acid, and vanillic acid, Table 4) also showed similarities to the results obtained for apple peel samples (44). Furthermore, quantified contents of polyphenolic compounds (Table 4) were in the same order of magnitude as the results that various authors published for apple samples (20, 41, 42, 44–46). Comparing the same apple varieties (“Franskar,” “Fuhr,” “Furuholm,” and “Løeple”), cultivated in different areas, yielded higher quantified values of polyphenolic compounds in samples from the NIBIO Ullensvang area than from Njøs (Table 4).

With UHPLC-LTQ OrbiTrap MS analysis performed on nine indigenous apple samples from the NIBIO Ullensvang area (Table 5), 47 phenolic compounds were identified, of which 23 phenolic acids and their derivatives, along with 24 flavonoids and their derivatives. Moreover, more glycosides have been identified (Table 5) than previously quantified by UHPLC-DAD MS/MS (Table 4). Their fragmentation pathway showed occurring the MS^2^, MS^3^, and MS^4^ fragments (Table 5). Deficiency of MS^4^ fragments was observed for several compounds (12 phenolic acids and derivatives, and 2 flavonoids), but still MS^4^ fragments were presented in most polyphenolic compounds. Of all identified compounds, 31 polyphenolic compounds were confirmed in all nine apple samples (17 flavonoids and derivatives, and 14 phenolic acids and derivatives; Table 5). Among four identified favan-3-ols, epicatechin and B-type proanthocyanidin isomer 2 were confirmed in all nine apple samples. Nevertheless, identified flavanols (epicatechin, catechin, and B type proanthocyanidins) were found in many apple samples published by other authors (41, 42, 45, 46, 59). These compounds increase the antioxidant potential of samples, as was noted that flavanol-3-ols, moreover procyanidins, present higher antioxidants than other flavonoids (60). In addition, other authors (20) showed a high correlation between antioxidant activity and flavan-3-ols, known as high radical scavengers. Polyphenolic profiles of nine indigenous apple samples (Table 5) showed the most similarities between samples no. 15 “Fuhr” and no. 72 “Vinterrosenstips” (only one difference between them was found, presence of *p*-hydroxyphenylacetic acid in no. 72, Table 5). The next pairs of apples that showed similarity (with three differences between them) were no. 47 “Raud Gravenstein” and no. 68 “Tveiteple”; no. 68 “Tveiteple” and no. 72 “Vinterrosenstips”; no. 69 “Ulgenes” and no. 71 “Vanleg Torstein” (Table 5).

**Table 5:** Phenolic profile of nine Norwegian apple samples from the NIBIO Ullensvang area (sample no. 15 “Furuholm,” “Fuhr,” no. 30 “Kaupanger,” no. 44 “Prins,” no. 47 “Raud Gravenstein,” no. 66 “Tormodseple,” no. 68 “Tveiteple,” no. 69 “Ulgenes,” no. 71 “Vanleg Torstein,” and no. 72 “Vinterrosenstips”).

***t***_**R**_ **, min**	**Compound name**	**Molecular formula, [M–H]^**−**^**	**Calculated mass, [M–H]^**−**^**	**Exact mass, [M–H]^**−**^**	**Δ ppm**	**MS^**2**^ fragments, (% base peak)**	**MS^**3**^ fragments, (% base peak)**	**MS^**4**^ fragments, (% base peak)**	**15**	**30**	**44**	**47**	**66**	**68**	**69**	**71**	**72**	**References**
4.10	**Protocatechiuic acid** ***O*****-hexoside**	C_13_H_15_O${\text{O}}_{9}^{-}$	315.07216	315.07178	1.18	108 (9), 109 (12), 151 (8), 152 (49), **153** (100), 163 (9), 165 (13)	108 (22), **109** (100)	81 (63), **123** (100)	**+**	**+**	**+**	**+**	**–**	**+**	**+**	**+**	**+**	(47)
4.22	**Gallic acid** *^***a***^*	C_7_H_5_${\text{O}}_{5}^{-}$	169.01425	169.01398	1.60	**125** (100)	**107** (100)	NR	**+**	**+**	**+**	**+**	**+**	**+**	**+**	**+**	**+**	
4.56	**Protocatechiuic acid** *^***a***^*	C_7_H_5_${\text{O}}_{4}^{-}$	153.01933	153.01893	2.65	**109** (100), 110 (5)	**65** (100), 81 (71)	NR	**+**	**+**	**+**	**+**	**+**	**+**	**+**	**+**	**+**	
4.80	**3-** ***O*** **-Caffeoylquinic acid** *^***a***^*	C_16_H_17_${\text{O}}_{9}^{-}$	353.08781	353.08727	1.51	135 (7), 179 (32), **191** (100), 192 (4)	85 (92), 109 (21), 111 (47), **127** (100), 171 (26), 173 (72)	81 (16), **85** (100), 95 (16), 99 (15), 109 (12)	**+**	**–**	**+**	**+**	**+**	**+**	**–**	**–**	**+**	
4.96	**Caffeic acid hexoside**	C_15_H_17_${\text{O}}_{9}^{-}$	341.08781	341.08768	0.36	135 (9), 147 (10), 161 (42), **179** (100), 180 (6), 203 (8), 281 (4)	**135** (100)	91 (12), **107** (100), 117 (30)	**+**	**+**	**+**	**+**	**+**	**+**	**+**	**+**	**+**	(48)
5.07	**5-** ***O*** **-Caffeoylquinic acid** *^***a***^*	C_16_H_17_${\text{O}}_{9}^{-}$	353.08781	353.08754	0.74	179 (6), **191** (100), 192 (5), 305 (5), 315 (3)	**85** (100), 93 (49), 111 (25), 127 (69), 171 (29), 173 (62)	NR	**+**	**+**	**–**	**+**	**+**	**+**	**–**	**–**	**+**	
5.10	**B type proanthocyanidin isomer 1**	C_30_H_25_${\text{O}}_{12}^{-}$	577.13515	577.13293	3.85	287 (8), 289 (24), 407 (53), **425** (100), 426 (8), 451 (26), 559 (8)	273 (7), 381 (6), **407** (100)	281 (94), 283 (37), **285** (100), 297 (36), 389 (35)	**–**	**+**	**–**	**–**	**–**	**–**	**+**	**+**	**–**	(49)
5.44	**5-** ***O*** **-Caffeoylquinic acid isomer**	C_16_H_17_${\text{O}}_{9}^{-}$	353.08781	353.08751	0.84	179 (3), **191** (100)	85 (94), 93 (58), 111 (36), **127** (100), 171 (26), 173 (72)	81 (5), 83 (20), **85** (100), 99 (33), 109 (24)	**+**	**+**	**+**	**+**	**+**	**+**	**+**	**+**	**+**	(50, 51)
5.49	**Catechin** *^***a***^*	C_15_H_13_${\text{O}}_{6}^{-}$	289.07176	289.07106	2.44	179 (10), 203 (8), 205 (33), 231 (6), **245** (100), 246 (7), 247 (5)	161 (19), 175 (11), 187 (23), 188 (15), **203** (100), 227 (28)	157 (13), 161 (29), **175** (100), 185 (18), 188 (58)	**+**	**+**	**–**	**+**	**–**	**–**	**+**	**+**	**+**	
5.54	***p*** **-Coumaric acid hexoside**	C_15_H_17_${\text{O}}_{8}^{-}$	325.09289	325.09283	0.19	119 (9), **145** (100), 163 (76), 187 (43), 205 (8), 265 (15), 289 (48)	**117** (100)	NR	**+**	**+**	**+**	**+**	**+**	**+**	**+**	**+**	**+**	(48)
5.57	**3-** ***O*** **-** ***p*** **-Coumaroylshikimic acid**	C_16_H_15_${\text{O}}_{7}^{-}$	319.08233	319.08224	0.26	119 (10), 137 (9), 139 (6), **145** (100), 146 (8), 257 (5), 275 (6)	**117** (100)	NR	**+**	**–**	**+**	**+**	**+**	**+**	**–**	**+**	**+**	(52)
5.68	**B type proanthocyanidin isomer 2**	C_30_H_25_${\text{O}}_{12}^{-}$	577.13515	577.13373	2.45	287 (6), 289 (17), 407 (46), **425** (100), 426 (7), 451 (19), 559 (5)	273 (7), 381 (5), **407** (100)	281 (73), 283 (37), 285 (100), 297 (31), 389 (27)	**+**	**+**	**+**	**+**	**+**	**+**	**+**	**+**	**+**	(49)
5.83	**Ferulic acid hexoside**	C_16_H_19_${\text{O}}_{9}^{-}$	355.10346	355.10282	1.80	134 (4), 160 (4), 175 (33), **193** (100), 217 (39), 235 (6), 295 (4)	**134** (100), 149 (31), 178 (17)	**106** (100)	**+**	**+**	**+**	**+**	**+**	**+**	**+**	**+**	**+**	(48)
5.96	**Caffeic acid** *^***a***^*	C_9_H_7_${\text{O}}_{4}^{-}$	179.03498	179.03485	0.76	89 (16), 113 (5), 119 (6), **135** (100), 136 (6), 143 (13), 161 (9)	**91** (100), 93 (22), 107 (99), 117 (6)	NR	**+**	**+**	**+**	**+**	**+**	**+**	**+**	**+**	**+**	
6.00	**Epicatechin** *^***a***^*	C_15_H_13_${\text{O}}_{6}^{-}$	289.07176	289.07127	1.69	179 (9), 203 (8), 205 (31), 231 (4), **245** (100), 246 (7), 247 (4)	161 (18), 175 (10), 187 (22), 188 (13), **203** (100), 227 (25)	161 (38), 174 (24), **175** (100), 185 (25), 188 (80)	**+**	**+**	**+**	**+**	**+**	**+**	**+**	**+**	**+**	
6.06	**4-** ***O*** **-** ***p*** **-Coumaroylquinic acid**	C_16_H_17_${\text{O}}_{8}^{-}$	337.09289	337.09266	0.68	163 (6), **173** (100)	71 (19), **93** (100), 109 (8), 111 (54), 155 (14)	NR	**+**	**+**	**+**	**+**	**+**	**+**	**+**	**+**	**+**	(53, 54)
6.07	***p*** **-Coumaric acid** *^***a***^*	C_9_H_7_${\text{O}}_{3}^{-}$	163.04007	163.03986	1.28	101 (33), 113 (15), **119** (100), 120 (13), 131 (48), 133 (32), 143 (21)	78 (15), **91** (100)	NR	**+**	**+**	**+**	**+**	**+**	**+**	**+**	**+**	**+**	
6.12	**3-** ***O*** **-Caffeoylshikimic acid**	C_16_H_15_${\text{O}}_{8}^{-}$	335.07724	335.07721	0.09	135 (25), 161 (3), **179** (100), 180 (7)	**135** (100)	**79** (100), 107 (16), 108 (35), 117 (6)	**+**	**–**	**–**	**–**	**+**	**–**	**–**	**+**	**+**	(55)
6.15	***p*** **-Hydroxybenzoic acid** *^***a***^*	C_7_H_5_${\text{O}}_{3}^{-}$	137.02442	137.02436	0.44	109 (8), **93** (100)	**93** (100)	NR	**+**	**+**	**+**	**+**	**+**	**+**	**+**	**+**	**+**	
6.34	***p*** **-Hydroxyphenylacetic acid** *^***a***^*	C_8_H_7_${\text{O}}_{3}^{-}$	151.04007	151.03998	0.60	121 (14), **107** (100), 95 (70), 79 (16), 59 (23)	123 (10), 95 (32), **79** (100), 69 (10), 51 (18)	**108** (100)	**–**	**+**	**+**	**–**	**–**	**–**	**–**	**–**	**+**	
6.35	**Methyl 3-** ***O*** **-caffeoylquinate**	C_17_H_19_${\text{O}}_{9}^{-}$	367.10346	367.10297	1.31	133 (6), 135 (22), **161** (100), 162 (7), 179 (3), 193 (3), 335 (3)	**133** (100)	**105** (100)	**+**	**+**	**+**	**+**	**+**	**+**	**+**	**+**	**+**	(56, 57)
6.46	**Aesculetin** *^***a***^*	C_9_H_5_${\text{O}}_{4}^{-}$	177.01933	177.01923	0.56	149 (8), **133** (100), 105 (12), 89 (4)	**89** (100)	NR	**–**	**–**	**–**	**–**	**–**	**–**	**–**	**–**	**–**	
6.58	**Methyl 5-** ***O*** **-caffeoylquinate**	C_17_H_19_${\text{O}}_{9}^{-}$	37.10346	367.10313	0.88	134 (3), 135 (44), 136 (4), 161 (11), **179** (100), 180 (9), 191 (20)	**135** (100)	79 (57), 91 (19), 106 (57), **107** (100), 117 (10)	**+**	**+**	**+**	**+**	**–**	**+**	**+**	**+**	**+**	
6.65	**Syringic acid** *^***a***^*	C_9_H_9_${\text{O}}_{5}^{-}$	197.04555	197.04550	0.25	**183** (100), 153 (41), 138 (10)	**167** (100), 138 (9), 123 (5)	NR	**–**	**+**	**+**	**+**	**–**	**–**	**–**	**+**	**–**	
6.67	**3-Hydroxyphloretin 2'-** ***O*** **-pentosyl-(1–6)-hexoside**	C_26_H_31_${\text{O}}_{15}^{-}$	583.16684	583.16461	3.83	167 (3), 271 (5), **289** (100)	123 (3), 125 (40), **167** (100), 245 (17), 271 (71)	**123** (100), 125 (14)	**+**	**+**	**+**	**+**	**+**	**+**	**+**	**+**	**+**	(49)
6.78	**Quercetin 3-** ***O*** **-glucoside** *^***a***^*	C_21_H_19_${\text{O}}_{12}^{-}$	463.08820	463.08597	4.82	300 (37), **301** (100), 302 (10)	151 (75), **179** (100), 255 (31), 257 (12), 271 (46), 272 (18)	**151** (100)	**+**	**+**	**+**	**+**	**+**	**+**	**+**	**+**	**+**	
6.91	**Methyl 3-** ***O*** **-** ***p*** **-coumaroylquinate**	C_17_H_19_${\text{O}}_{8}^{-}$	351.10854	351.10794	1.71	117 (5), 119 (9), **145** (100), 146 (6), 163 (3)	**117** (100)	NR	**+**	**+**	**+**	**+**	**+**	**+**	**+**	**+**	**+**	(5)
6.93	**Rutin** *^***a***^*	C_27_H_29_${\text{O}}_{16}^{-}$	609.14611	609.14592	0.31	**301** (100)	**179** (100), 151 (78), 107 (4)	**151** (100), 107 (2)	**+**	**+**	**+**	**+**	**+**	**+**	**+**	**+**	**+**	
7.04	**Quercetin 3-** ***O*** **-pentoside isomer 1**	C_20_H_17_${\text{O}}_{11}^{-}$	433.07764	433.07641	2.83	300 (15), **301** (100), 302 (10)	151 (79), **179** (100), 255 (18), 271 (18), 273 (19), 283 (16)	**151** (100)	**+**	**+**	**+**	**+**	**+**	**+**	**+**	**+**	**+**	(49)
7.14	**Phloretin 2'-** ***O*** **-pentosyl-(1–6)-hexoside**	C_26_H_31_${\text{O}}_{14}^{-}$	567.17193	567.16998	3.44	167 (5), **273** (100), 274 (10)	123 (4), 125 (3), **167** (100)	**123** (100), 125 (13), 151 (3)	**+**	**+**	**+**	**+**	**+**	**+**	**+**	**+**	**+**	(49)
7.16	**3-Hydroxyphloretin**	C_15_H_13_${\text{O}}_{6}^{-}$	289.07176	289.07147	1.00	125 (39), **167** (100), 245 (19), 271 (74)	**123** (100), 125 (14), 151 (3)	NR	**+**	**+**	**–**	**+**	**+**	**+**	**+**	**+**	**+**	(58)
7.22	**Quercetin 3-** ***O*** **-pentoside isomer 2**	C_20_H_17_${\text{O}}_{11}^{-}$	433.07764	433.07756	0.18	300 (11), **301** (100), 302 (11)	151 (78), **179** (100), 255 (12), 271 (9), 273 (18), 283 (14)	**151** (100)	**+**	**+**	**–**	**+**	**+**	**+**	**+**	**+**	**+**	(49)
7.32	**Quercetin 3-** ***O*** **-rhamnoside** *^***a***^*	C_21_H_19_${\text{O}}_{11}^{-}$	447.09329	447.09319	0.21	300 (20), **301** (100), 302 (8)	151 (77), **179** (100), 255 (29), 271 (41), 273 (20), 283 (21)	**151** (100)	**+**	**+**	**+**	**+**	**+**	**+**	**+**	**+**	**+**	
7.37	**Isorhamnetin 3-** ***O*** **-rutinoside** *^***a***^*	C_28_H_31_${\text{O}}_{16}^{-}$	623.16176	623.16173	0.05	**315** (100), 300 (20), 271 (9), 255 (6)	**300** (100), 287 (5), 272 (4)	**271** (100), 255 (50), 151 (4)	**+**	**+**	**+**	**+**	**+**	**+**	**+**	**+**	**+**	
7.43	**Vanillic acid** *^***a***^*	C_8_H_7_${\text{O}}_{4}^{-}$	167.03498	167.03492	0.36	153 (10), 152 (79), 124 (11), **123** (100), 108 (222)	**108** (100)	123 (29), 80 (35), **78** (100)	**+**	**+**	**+**	**+**	**+**	**+**	**+**	**+**	**+**	
7.50	**Naringin** *^***a***^*	C_27_H_31_${\text{O}}_{14}^{-}$	579.17193	579.17145	0.83	**459** (100), 357 (5), 313 (26), 271 (44), 235 (12)	441 (30), **357** (100), 339 (31), 271 (54), 235 (87)	**339** (100), 169 (22), 151 (50), 125 (21)	**+**	**–**	**+**	**+**	**+**	**+**	**–**	**–**	**+**	
7.56	**Sinapic acid** *^***a***^*	C_11_H_11_${\text{O}}_{5}^{-}$	223.06120	223.06118	0.09	**208** (100), 179 (31), 164 (20)	193 (10), **164** (100), 149 (14), 135 (3)	**149** (100), 135 (33)	**+**	**–**	**+**	**–**	**–**	**+**	**–**	**–**	**+**	
7.65	**Isorhamnetin 3-** ***O*** **-glucoside** *^***a***^*	C_22_H_21_${\text{O}}_{12}^{-}$	477.10385	477.10208	3.71	357 (21), 315 (48), **314** (100), 300 (5), 285 (10), 271 (11)	300 (30), **285** (100), 271 (73), 257 (8), 243 (24)	**270** (100)	**+**	**+**	**+**	**+**	**+**	**+**	**+**	**+**	**+**	
7.65	**Kaempferol 7-** ***O*** **-glucoside** *^***a***^*	C_21_H_19_${\text{O}}_{11}^{-}$	447.09329	447.09192	3.06	327 (18), 285 (80), **284** (100), 255 (9)	**255** (100), 227 (9)	**227** (100), 211 (61)	**+**	**+**	**+**	**+**	**+**	**+**	**+**	**+**	**+**	
7.67	**Phloretin 2′-*****O*****-glucoside (Phloridzin)** *^***a***^*	C_21_H_23_${\text{O}}_{10}^{-}$	435.12967	435.12909	1.34	**273** (100), 274 (7)	123 (4), 125 (3), **167** (100)	**123** (100), 125 (13), 151 (3)	**+**	**+**	**+**	**+**	**+**	**+**	**+**	**+**	**+**	
7.67	**Phloretin** *^***a***^*	C_15_H_13_${\text{O}}_{5}^{-}$	273.07685	273.07674	0.39	123 (3), 125 (3), **167** (100), 168 (6)	**123** (100), 125 (12), 151 (3)	**81** (100), 95 (64), 97 (4), 105 (3), 108 (5)	**+**	**+**	**+**	**+**	**+**	**+**	**+**	**+**	**+**	
7.58	**Ferulic acid** *^***a***^*	C_10_H_9_${\text{O}}_{4}^{-}$	193.05063	193.05062	0.05	178 (72), **149** (100), 134 (39)	**134** (100)	NR	**+**	**+**	**+**	**+**	**+**	**+**	**+**	**+**	**+**	
8.90	**Quercetin** *^***a***^*	C_15_H_9_${\text{O}}_{7}^{-}$	301.03538	301.03479	1.94	151 (46), **179** (100), 180 (4), 193 (3), 257 (6), 271 (5), 273 (8)	**151** (100)	63 (5), 65 (3), 83 (13), **107** (100)	**+**	**+**	**+**	**+**	**+**	**+**	**+**	**+**	**+**	
9.05	**Eriodictyol** *^***a***^*	C_15_O_11_${\text{O}}_{6}^{-}$	287.05611	287.05533	2.72	**151** (100), 107 (8)	**107** (100)	**65** (100)	**+**	**+**	**+**	**+**	**+**	**+**	**+**	**+**	**+**	
9.93	**Naringenin** *^***a***^*	C_15_H_11_${\text{O}}_{5}^{-}$	271.06120	271.05989	4.83	225 (5), 177 (11), **151** (100)	**107** (100)	**65** (100)	**+**	**+**	**+**	**+**	**+**	**+**	**+**	**+**	**+**	
10.11	**Kaempferol** *^***a***^*	C_15_H_9_${\text{O}}_{6}^{-}$	285.04046	285.03979	2.35	**255** (100), 227 (10)	**211** (100), 195 (4), 167 (16)	211 (41), **137** (100)	**+**	**+**	**+**	**+**	**+**	**+**	**+**	**+**	**+**	
12.21	**Acacetin** *^***a***^*	C_16_H_11_${\text{O}}_{5}^{-}$	283.06119	283.06033	3.04	**268** (100)	**268** (100), 240 (29)	239 (13), 223 (18), 211 (100), 196 (74), 172 (62)	**+**	**–**	**+**	**+**	**+**	**+**	**–**	**–**	**+**	

*^**a**^Confirmed using standards, while the other compounds were identified by evaluation of its HRMS and MS^n^ data (see references in the table)*.

*“NR,” not recorder; “+,” present compound; “**–**,” not present compound. The bold values refer to the most intense ions*.

Relying on the previously described differences between apple samples from two areas (observed from the quantification of polyphenols, Table 4), confirmed differences could be noted from their polyphenolic profiles (Table 5). The observations that differentiate these apple samples are non-appearance of aesculetin, and the finding eriodictyol, naringenin, gallic acid, and vanillic acid in apple samples from the NIBIO Ullensvang area (Tables 4, 5).

#### Determination of TPC and RSA

Antioxidant activity expressed as TPC and RSA (Table 4) showed opposite results from the described quantification of phenolic compounds. The mean value for RSA was higher for the Njøs apples (66.57 in contrast to 39.18 mol TE/kg dw for NIBIO samples), while the TPC values were similar (9.00 and 8.43 g GAE/kg dw, respectively). The maximum TPC and RSA values for apples from Njøs were in the same sample (no. 94 “Leriseple”), while contrary to this, the Pearson coefficient showed very low independence between TPC and RSA for investigated apples from both areas (0.18 for NIBIO and 0.35 for Njøs samples). Considering the qualitative phenolic analysis showed a greater impact on the antiproliferative activity of apples than the quantitative analysis (46), a similar observation could be noted for antioxidant activity in this study. Most of the polyphenolic compounds showed maximum values in samples from the NIBIO Ullensvang area, but the TPC and RSA values did not follow that observation (Table 4). However, results of TPC were similar to those published by other authors (43). As suggested by other authors (61), apple samples showed antioxidant activities that increase the importance of isolating valuable compounds that affect these activities. The dependence of antioxidant activity on apple varieties, observed in this study, was in accordance with the data from the literature (20).

### Statistical Analysis

#### Statistics Applied on the Results of Sugar Compounds

**Figure 2:**
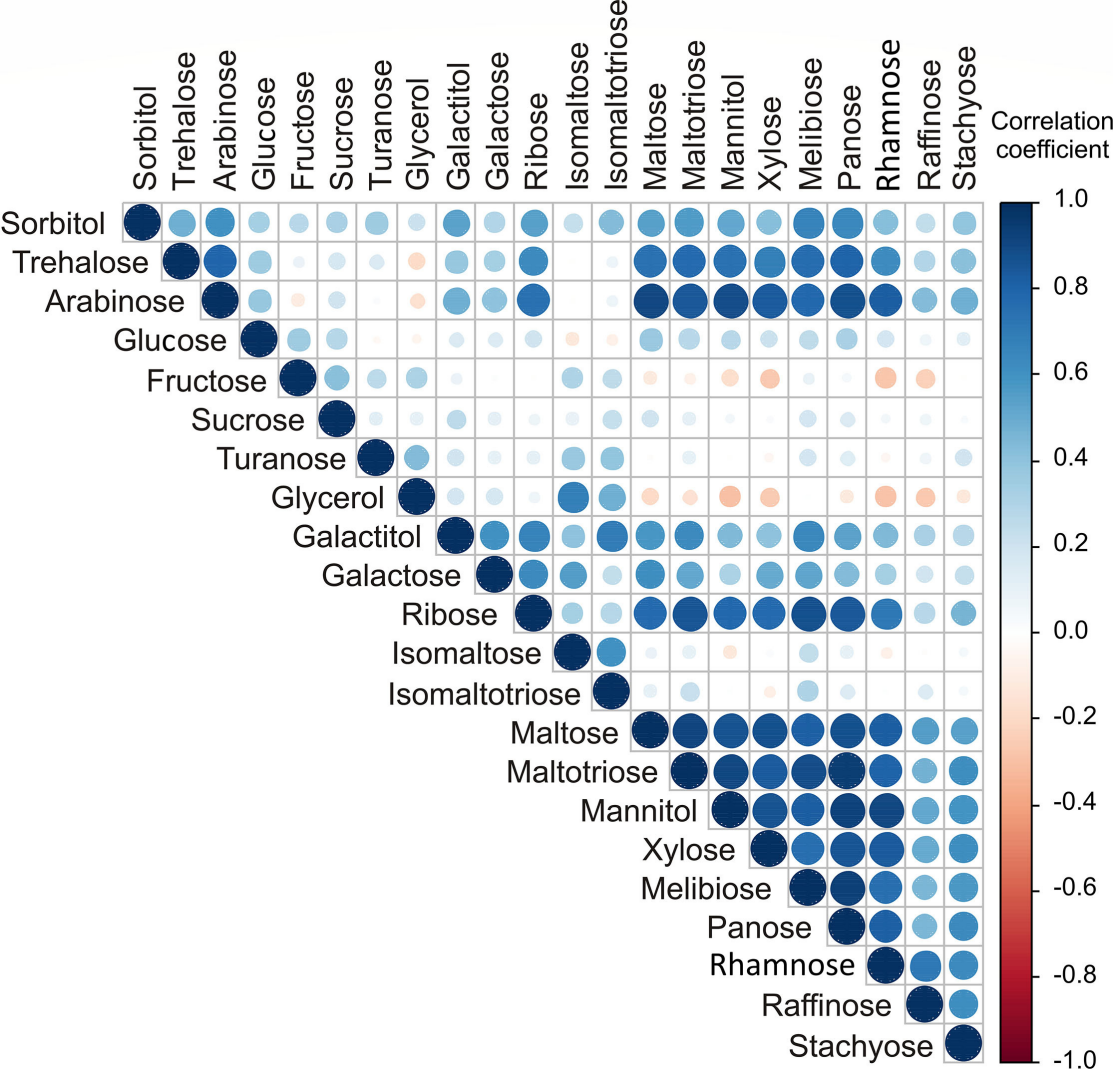
Color correlation graph between sugar content in apple samples.

The highest positive correlations for sugar content (Figure 2) were found between arabinose and maltose, maltotriose, mannitol, panose, and rhamnose (*r* = 0.902; *r* = 0.846; *r* = 0.885; *r* = 0.874; *r* = 0.823, respectively), trehalose, and panose (*r* = 0.803). The positive correlations between contents of maltose, maltotriose, mannitol, xylose, melibiose, panose, and rhamnose were found, with high correlation coefficients in the range between 0.756 and 0.944.

The PCA of the sugar content in apple samples (Supplementary Figure S1) explained that the first three principal components summarized 70.90% of the total variance in the 22 parameters (sorbitol, trehalose, arabinose, glucose, fructose, sucrose, turanose, glycerol, galactitol, galactose, ribose, isomaltose, isomaltotriose, maltose, maltotriose, mannitol, xylose, melibiose, panose, rhamnose, raffinose, and stachyose). According to the results of the PCA, the content of arabinose (which contributed 7.9% of the total variance, based on correlations), ribose (7.4%), maltose (8.5%), maltotriose (8.8%), and mannitol (8.1%) showed negative influence on PC1. In contrast, the content of fructose (9.3%), turanose (9.1%), glycerol (18.3%), isomaltose (18.3%), and isomaltotriose content (16.7%) positively influenced the calculation of PC2 (Supplementary Figure S1). The content of glucose (28.4%) and sucrose (17.7%) positively affected the third principal component (PC3).

The results of the applied statistical analysis on sugar compounds showed that apple samples from the NIBIO Ullensvang area and Njøs differ mainly in the content of galactitol, sorbitol, galactose, trehalose, and arabinose.

#### Statistics Applied on the Results of Organic Acids

**Figure 3:**
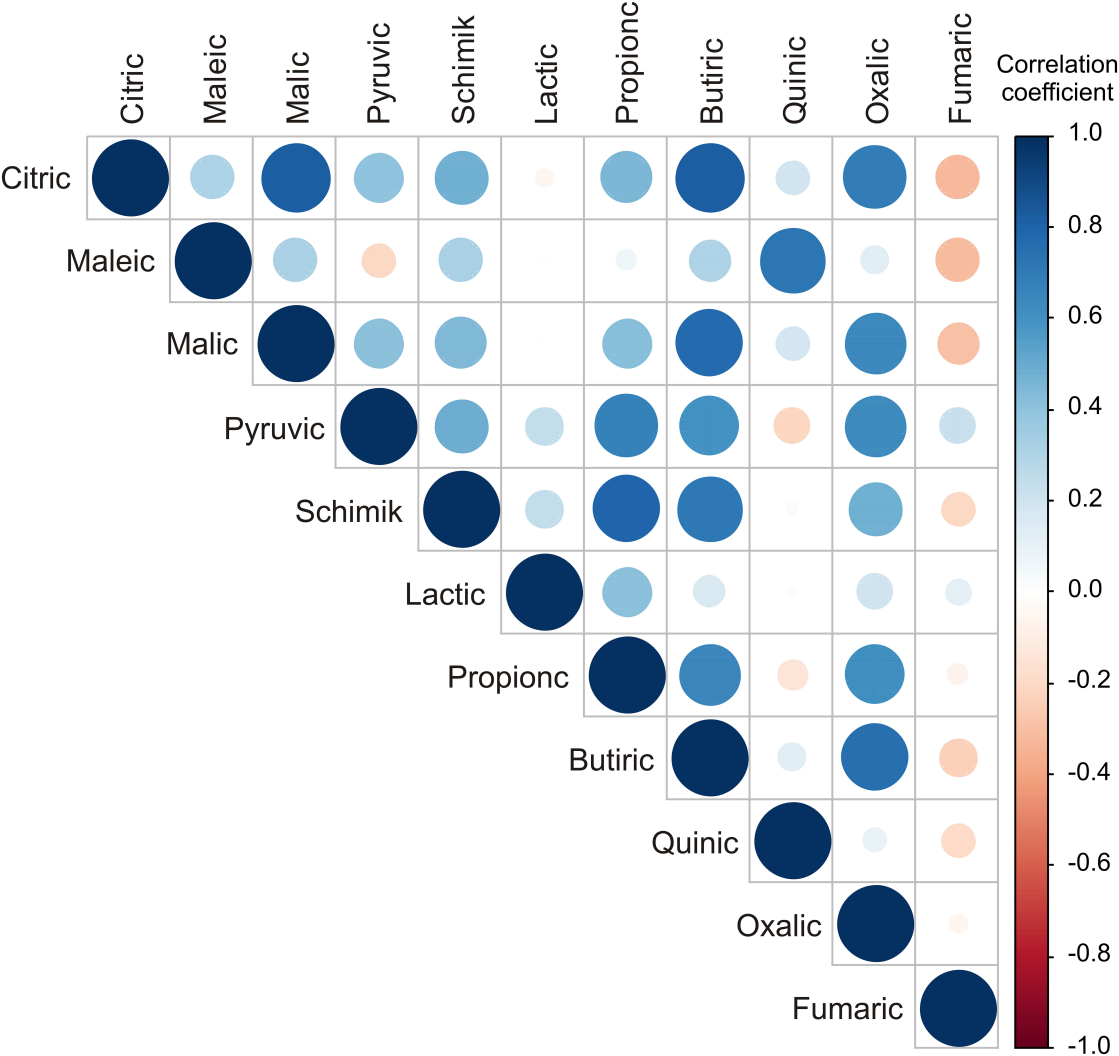
Color correlation graph between fruit acids content in apple samples.

The highest positive correlations in organic acid contents were found between citric and malic and oxalic acids content (*r* = 0.825; *r* = 0.692, respectively), maleic and quinic acids content (*r* = 0.724), malic and butyric (*r* = 0.774), pyruvic and propionic (*r* = 0.678), shikimic and propionic and butyric (*r* = 0.792 and *r* = 0.720, respectively), and propionic and butyric and oxalic acids content (*r* = 0.880 and *r* = 0.615, respectively; Figure 3).

The PCA of the organic acids in apple samples (Supplementary Figure S2) explained that the first three principal components outlined 75.28% of the total variance in the 11 parameters (citric, maleic, malic, pyruvic, shikimic, lactic, propionic, butyric, quinic, oxalic, and fumaric). The content of citric (14.3% of the total variance, depending on correlations), malic (13.3%), pyruvic (9.3%), shikimic (12.6%), propionic (12.8%), butyric (18.3%), and oxalic acids content influenced negatively to PC1 calculation. The content of fruit acid such as pyruvic (14.5% of the total variance, depended on correlations) and fumaric (13.7%), influenced positively to the PC2 coordinate, while the content of maleic (27.4%) and quinic (26.7%) influenced negatively to the PC2 coordinate (Supplementary Figure S2). The content of fruit acid such as maleic (12.2% of the total variance, depended on correlations), lactic (46.5%), and quinic (11.1%) affected positively to PC3 calculation (Supplementary Figure S2), while the content of malic acid (7.4%) influenced negatively to PC3 coordinate.

From the applied statistical analysis results on the organic acids content, the samples from the NIBIO Ullensvang area and Njøs differed the most in the citric and butyric acids content.

#### Statistics Applied on the Results of Minerals

**Figure 4:**
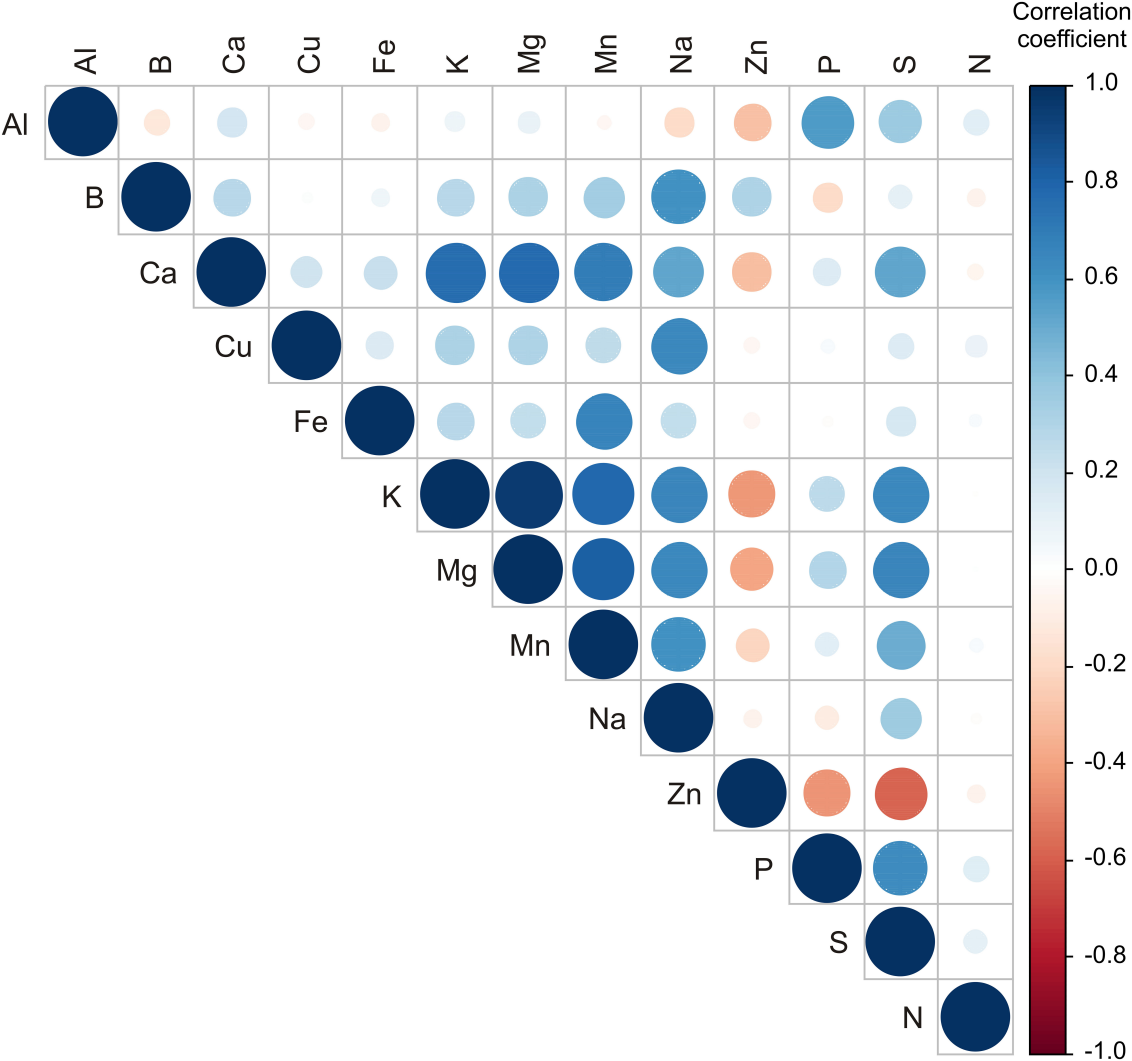
Color correlation graph between metal content in apple samples.

Statistically significant correlations (*p* ≤ 0.05) were found between several element contents in the samples (Figure 4). The circle's color is defined by the correlation coefficients value, while the circle's size is defined by the *p*-value of the correlation. The content of Mn was positively correlated to Fe, K, Mg content (*r* = 0.664; *r* = 0.790; and *r* = 0.814, respectively). The content of Na was positively correlated to K, Mg, and Mn content (*r* = 0.652; *r* = 644; and *r* = 0.604, respectively). The content of S was positively correlated to K, Mg, and P content (*r* = 0.647; *r* = 0.657; and *r* = 0.628, respectively). Similar results for correlation between Mg and S, K and S, and between S and P, were determined in “Honeycrisp” apples grown on different rootstocks in Champlain valley in New York and Western New York climatic conditions (62, 63).

The PCA of the metal content in samples explained that the first three principal components outlined 66.37% of the total variance in the 13 parameters (Al, B, Ca, Cu, Fe, K, Mg, Mn, Na, Zn, P, S, and N). According to the results of the PCA, the content of Ca (which contributed 13.1% of the total variance, based on correlations), K (17.3%), Mg (17.5%), Mn (14.7%), Na (10.5%), and S (11.1) exhibited positive influence to the first principal component (PC1). The content of Al (20.1% of the total variance, based on correlations), P (24.2%), and S (8.6%) showed a positive influence on the second principal component (PC2), while B (12.7%), Na (10.8%), and Zn content (15.3%) exerted a negative score according to PC2 component. B content (16.0% of the total variance, based on correlations) showed a positive influence on the third principal component (PC3) calculation, while the content of Cu (24.7%), Fe (18.0%), and N content (31.8%) exerted a negative influence to PC3 (Supplementary Figure S3).

The samples from the NIBIO Ullensvang area and Njøs differ mainly in the content of Al, P, S, and Zn. Statistical analysis shows noticeable differences for apple samples from these two areas. As the higher content of macronutrients was already noted in samples from the NIBIO Ullensvang area (Table 3), PCA confirmed these differences (Supplementary Figure S3).

#### Statistics Applied on the Results of Phenolics, TPC, and RSA

**Figure 5:**
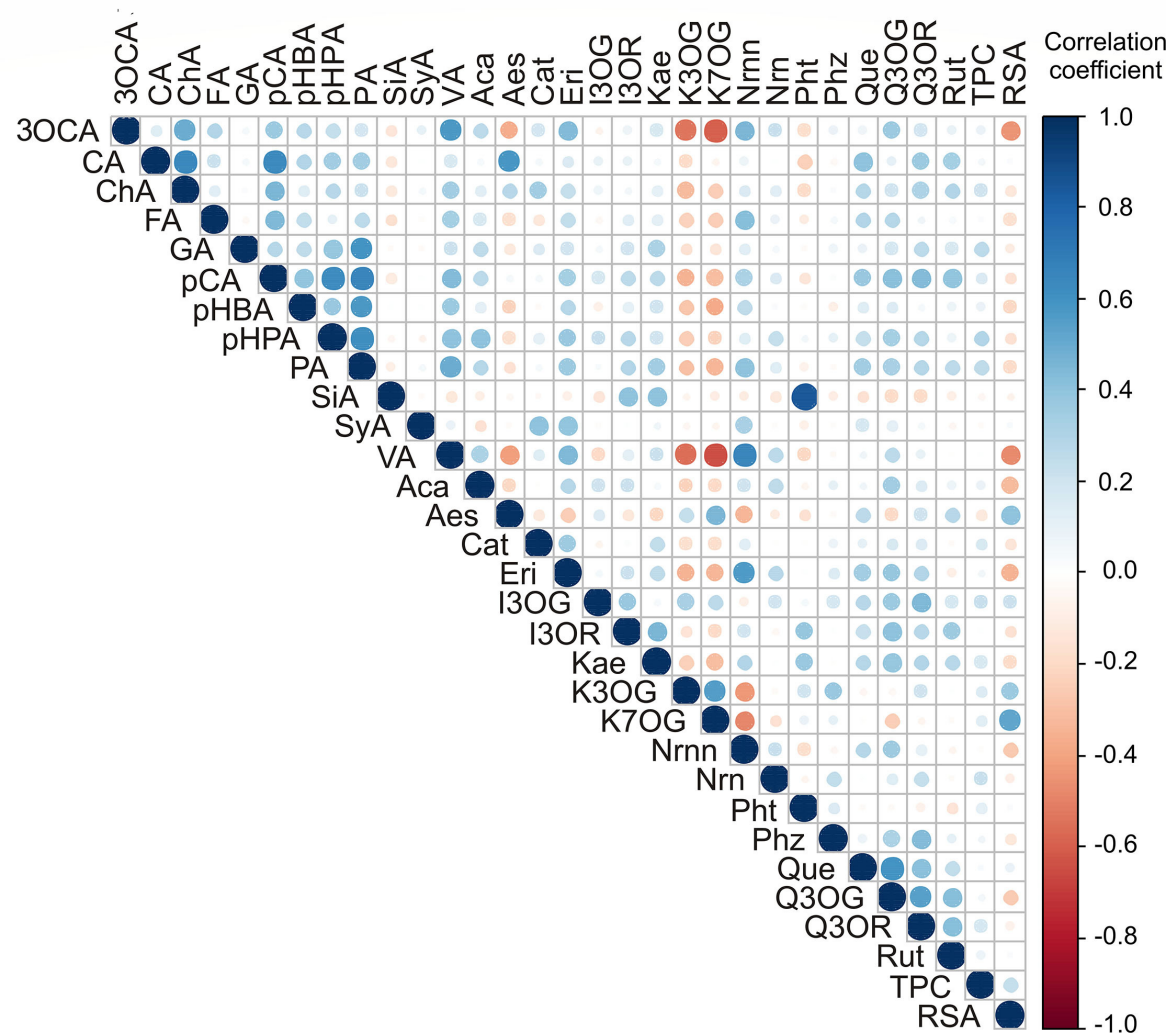
Color correlation graph between phenolic content in apple samples (3OCA, 3-*O*-caffeoylquinic acid; CA, caffeic acid; ChA, chlorogenic acid; FA, ferulic acid; GA, gallic acid; pCA, *p*-coumaric acid; pHBA, *p*-hydroxybenzoic acid; pHPA, *p*-hydroxyphenylacetic acid; PA, protocatechuic acid, SiA, sinapic acid; SyA, syringic acid; VA, vanillic acid; Aca, acacetin; Aes, aesculetin; Cat, catechin; Eri, eriodictyol; I3OG, isorhamnetin 3-*O*-glucoside; I3OR, isorhamnetin 3-*O*-rutinoside; Kae, kaempferol; K3OG, kaempferol 3-O-glucoside; K7OG, kaempferol 7-*O*-glucoside; Nrnn, naringenin; Nrn, naringin; Pht, phloretin; Phz, phlorizin; Que, quercetin; Q3OG, quercetin 3-*O-*glucoside; Q3OR, quercetin 3-*O-*rhamnoside; Rut, rutin).

Applied statistical analysis on the results of polyphenolic content and TPC and RSA is presented in Figure 5. The highest positive correlations were found between caffeic acid and chlorogenic acid (*r* = 0.646), *p*-coumaric acid and *p*-hydroxyphenylacetic acid, and protocatechuic acid (*r* = 0.622 and *r* = 0.650, respectively), *p*-hydroxyphenylacetic acid and protocatechuic acid (*r* = 0.615), naringenin and vanillic acid (r = 0.654), and quercetin and quercetin 3-*O-*glucoside (*r* = 0.599; Figure 5). In contrast, negative correlations were found between 3-*O*-caffeoylquinic acid and kaempferol 7-*O-*glucoside (*r* = −0.597), vanillic acid, and kaempferol 7-*O*-glucoside (*r* = −648).

The PCA of the content of polyphenolic compounds in apple samples (Supplementary Figure S4) showed that the first three principal components summarized 43.02% of the total variance in the 31 parameters (3-*O*-caffeoylquinic acid, caffeic acid, chlorogenic acid, ferulic acid, gallic acid, *p*-coumaric acid, *p*-hydroxybenzoic acid, *p*-hydroxyphenyl acetic acid, protocatechuic acid, sinapic acid, syringic acid, vanillic acid, acacetin, aesculetin, catechin, eriodictyol, isorhamnetin 3-*O*-glucoside, isorhamnetin 3-*O*-rutinoside, kaempferol, kaempferol 3-*O*-glucoside, kaempferol 7-*O*-glucoside, naringenin, naringin, phloretin, phlorizin, quercetin, quercetin 3-*O*-glucoside, quercetin 3-*O*-rhamnoside, and rutin). In addition, noted differences in the content of polyphenolic compounds for samples no. 81 and 83 (previously described in Section Determination of Polyphenolic Compounds and Polyphenolic Profile) were more obvious in PCA (Supplementary Figure S4). Furthermore, the content of *p*-coumaric acid (8.1% of the total variance, according to correlations), protocatechuic acid (7.5%), and vanillic acid (7.7%) exhibited a positive influence on the PC1 coordinate (Supplementary Figure S4). The content of aesculetin (11.6%), isorhamnetin 3-*O*-glucoside (10.7%), kaempferol 3-*O*-glucoside (7.1%), kaempferol 7-*O*-glucoside (7.4%), quercetin 3-*O*-glucoside (10.8%), and rutin (7.4%) showed a negative influence to PC2 coordinate computation (Supplementary Figure S4). In addition, the content of isorhamnetin 3-*O*-glucoside (12.8%) and kaempferol (11.5%) showed a positive influence on PC3 coordinate calculation.

According to the results of statistics, apple samples from the NIBIO Ullensvang area and Njøs differed mainly in kaempferol 3-*O*-glucoside, kaempferol 7-*O*-glucoside, RSA, vanillic acid, naringenin, 3-*O*-caffeoylquinic acid, and eriodyctol content. In addition, the PCA results (Figure 4, Supplementary Figure S4) complemented the previously observed differences between apples from these two areas. Moreover, these compounds could be used to differentiate Norwegian apple samples from different areas. The results were in accordance with the observations of other authors, which indicate the dependence of the chemical profile of apples on location (41) and variety (45).

## Conclusion

Through the extensive analysis that consisted of using modern analytical techniques and applied chemometrics, we analyzed 103 Norwegian apple varieties. The analyzed apple samples were differentiated by cultivation locations based on obtained results. Apples from the Ullensvang area showed higher nutritional values due to higher content of all detected sugars (744.54 g/kg dw), sugar alcohols (60.33 g/kg dw), organic acids (90.81 g/kg dw), and polyphenolic compounds (802.13 mg/kg dw) compared to those obtained for apples from Njøs. The most dominant minerals in all samples were potassium and zinc, but in the reverse order represented in samples from different areas. Moreover, applied statistical analysis showed a more detailed distinction between the apple cultivars of two Norwegian areas.

Due to the content of quantified polyphenolic compounds, as well as a significant number of identified polyphenolic compounds, these different apple cultivars could be treated as valuable health products. Given the observed antioxidant activity, apple samples could provide health benefits by preventing free radical-induced oxidative reactions. Overall, these results could be used for some further potential uses of Norwegian apples as an ingredient for functional foods or some following breeding programs. By using the distinctive UHPLC LTQ OrbiTrap MS technique, it was possible to determine polyphenolic profiles of nine selected indigenous apples, which provided additional parameters for the assessment of the apple's growing location. Besides, observed polyphenols in apple samples from Norway could be considered as potential markers for a more detailed assessment of apple's cultivation location.

## Data Availability Statement

The original contributions presented in the study are included in the article/Supplementary Material, further inquiries can be directed to the corresponding author.

## Author Contributions

Conceived and designed the analysis: ŽT, MM, and MFA. Sample distribution: MM and MFA. Performed the analysis: UG, TT, MN, BD, LP, and BL. Wrote the paper: MFA, ŽT, IĆ, LP, and MN. Conceived and designed the paper: ŽT, IĆ, and MN. All authors read and approved the final manuscript.

## Funding

This study was funded by the Research Council of Norway (project no. 280376).

## Conflict of Interest

The authors declare that the research was conducted in the absence of any commercial or financial relationships that could be construed as a potential conflict of interest.

## Publisher's Note

All claims expressed in this article are solely those of the authors and do not necessarily represent those of their affiliated organizations, or those of the publisher, the editors and the reviewers. Any product that may be evaluated in this article, or claim that may be made by its manufacturer, is not guaranteed or endorsed by the publisher.

## References

[1] Cornille A Giraud T Smulders MJ Roldán-Ruiz I Gladieux P. The domestication and evolutionary ecology of apples. Trends Genet. (2014) 30:57–65. 10.1016/j.tig.2013.10.002 24290193

[2] FaoStat. (2020). Available online at: http://www.fao.org/faostat/en/#data/QC (accessed on 30 April 2022).

[3] Fotirić Akšić M Lazarević K Šegan S Natić M Tosti T Cirić I. Meland M. Assessing the Fatty Acid, Carotenoid, and Tocopherol Compositions of Seeds from Apple Cultivars (*Malus domestica* Borkh) Grown in Norway. Foods. (2021) 10:1956. 10.3390/foods10081956 34441731 PMC8392653

[4] Alberti A dos Santos TPM Zielinski AAF dos Santos CME Braga CM Demiate IM. Impact on chemical profile in apple juice and cider made from unripe, ripe and senescent dessert varieties. LWT-Food Sci Technol. (2016) 65:436–43. 10.1016/j.lwt.2015.08.045

[5] Fotirić Akšić M Tosti T Nedić N Marković M Ličina V Milojković-Opsenica D. Tešić Ž. Influence of frost damage on the sugars and sugar alcohol composition in quince (*Cydonia oblonga* Mill) Floral nectar. Acta Physiol Plant. (2015) 37:1701. 10.1007/s11738-014-1701-y

[6] Oszmiański J Lachowicz S Gławdel E Cebulak T Ochmian I. Determination of phytochemical composition and antioxidant capacity of 22 old apple cultivars grown in Poland. Eur Food Res Technol. (2018) 244:647–62. 10.1007/s00217-017-2989-9

[7] Fotirić Akšić M Dabić Zagorac D Gašić U Tosti T Natić M Meland M. Analysis of apple fruit (*Malus* × *domestica* Borkh) quality attributes obtained from organic and integrated production systems. Sustainability. (2022) 14:5300. 10.3390/su14095300

[8] Wu J Gao H Zhao L Liao X Chen F Wang Z. Chemical compositional characterization of some apple cultivars. Food Chem. (2007) 103:88–93. 10.1016/j.foodchem.2006.07.030

[9] Zhang Y Li P Cheng L. Developmental changes of carbohydrates, organic acids, amino acids, and phenolic compounds in ‘Honeycrisp'apple flesh. Food Chem. (2010) 123:1013–8. 10.1016/j.foodchem.2010.05.053

[10] Lea AGH. Cider making. In: Lea AGH and Piggott JR, editors. Fermented Beverage Production. Glasgow: Blackie and Sons. (1995), p. 66–96. 10.1007/978-1-4757-5214-4_4

[11] Roussos PA Gasparatos D. Apple tree growth and overall fruit quality under organic and conventional orchard management. Sci Hortic. (2009) 123:247–52. 10.1016/j.scienta.2009.09.011

[12] Fotirić Akšić M Mutić J Tešić Ž Meland M. Evaluation of fruit mineral contents of two apple cultivars grown in organic and integrated production systems. Acta Hortic. (2020) 1281:59–66. 10.17660/ActaHortic.2020.1281.10

[13] Tagliavini M Zavalloni C Rombola AD Quartieri M Malaguti D Mazzanti F. Mineral nutrient partitioning to fruits of deciduous trees. Acta Hortic. (2000) 512:131–40. 10.17660/ActaHortic.2000.512.13

[14] Zavalloni C Marangoni B Tagliavini M Scudellari D. Dynamics of uptake of calcium, potassium and magnesium into apple fruit in high density planting. Acta Hortic. (2001) 564:113–21. 10.17660/ActaHortic.2001.564.12

[15] Fallahi E Mohan SK. Influence of nitrogen and rootstock on tree growth, precocity, fruit quality, leaf mineral nutrients, and fire blight in 'Scarlet Gala' apple. Hort Technology. (2000) 10:589–92. 10.21273/HORTTECH.10.3.589

[16] Gorinstein S Zachwieja Z Folta M Barton H Piotrowicz J Zemser M. Comparative contents of dietary fiber, total phenolics, and minerals in persimmons and apples. J Agr Food Chem. (2001) 49:952–7. 10.1021/jf000947k 11262055

[17] Hyson DA. A Comprehensive review of apples and apple components and their relationship to human health. Adv Nutr. (2011) 2:408–20. 10.3945/an.111.000513 22332082 PMC3183591

[18] Kim I Ku K-H Jeong M-C Kim SS Mitchell AE Lee J. comparison of the chemical composition and antioxidant activity of several new early- to mid-season apple cultivars for a warmer climate with traditional cultivars. J Sci Food Agric. (2019) 99:4712–24. 10.1002/jsfa.9712 30919973

[19] Krawitzky M Arias E Peiro JM Negueruela AI Val J Oria R. Determination of color, antioxidant activity, and phenolic profile of different fruit tissue of Spanish ‘Verde Doncella' apple cultivar. Int J Food Prop. (2014) 17:2298–311. 10.1080/10942912.2013.792829

[20] Carbone K Giannini B Picchi V Lo Scalzo R Cecchini F. Phenolic composition and free radical scavenging activity of different apple varieties in relation to the cultivar, tissue type and storage. Food Chem. (2011) 127:493–500. 10.1016/j.foodchem.2011.01.030 23140692

[21] Li Y Sun H Li J Qin S Niu Z Qiao X. Yang B. Influence of genetic background, growth latitude and bagging treatment on phenolic compounds in fruits of commercial cultivars and wild types of apples (*Malus* sp). Eur Food Res Technol. (2021) 247:1149–65. 10.1007/s00217-021-03695-0

[22] Aachary AA Eskin MNA. Bitterness in beverages. In: Aliani M, Eskin MNA, editors. The Bitterness: Perception, Chemistry and Food Processing. New York, NY: John Wiley&Sons, Incorporated. (2017), p. 81–103.

[23] Mikulic-Petkovšek M Stampar F. Veberic R. Parameters of inner quality of the apple scab resistant and susceptible apple cultivars (*Malus domestica Borkh). Sci Hortic*. (2007) 114:37–44. 10.1016/j.scienta.2007.05.004

[24] Bondonno NP Bondonno CP Ward NC Hodgson JM Croft KD. The cardiovascular health benefits of apples: Whole fruit vs. isolated compounds. Trends Food Sci Tech. (2017) 69:243–56. 10.1016/j.tifs.2017.04.012

[25] Muzher BM Younis RAA El-Halabi O Ismail OM. Genetic identification of some Syrian local apple (*Malus* sp.) cultivars using molecular markers. Res J Agric Biol Sci. (2007) 3:704–13.

[26] Pereira-Lorenzo S Fischer M Ramos-Cabrer AM Castro I. Apple (*Malus* spp.) breeding: present and future. In: Al-Khayri JM, Jain SM, Johnson DV, editors. Advances in Plant Breeding Strategies: Fruits, Vol. 3. Berlin: Springer International Publishing AG, part of Springer Nature. (2018), p. 3–30. 10.1007/978-3-319-91944-7_1

[27] Pereira-Lorenzo S Ramos-Cabrer AM Fischer M. Breeding apple (*Malus* × *domestica* Borkh). In: Jain SMP and Priyadarshan PM, editors. Breeding Plantation Tree Crops: Temperate Species. New York, NY: Springer (2009), p. 33–82. 10.1007/978-0-387-71203-1_2

[28] Pavlović AV Dabić DC Momirović NM Dojčinović BP Milojković-Opsenica DM. Tešić ŽLj, Natić MM. Chemical composition of two different extracts of berries harvested in Serbia. J Agric Food Chem. (2013) 61:4188–94. 10.1021/jf400607f 23600608

[29] Gašić UM Natić MM Mišić DM Lušić DV Milojković-Opsenica DM. Tešić ŽLj, Lušić D. Chemical markers for the authentication of unifloral *Salvia officinalis* L honey. J Food Compos Anal. (2015) 44:128–38. 10.1016/j.jfca.2015.08.008

[30] Taiz L Zeiger E. Plant physiology, 3rd ed. Sunderland, MA: Sinauer Associates Incorporated (2002).

[31] Liu Y Chen N Ma Z Che F Mao J Chen B. The changes in color, soluble sugars, organic acids, anthocyanins and aroma components in “Starkrimson” during the ripening period in China. Molecules. (2016) 21:812. 10.3390/molecules21060812 27338331 PMC6273148

[32] Durán-Soria S Pott DM Osorio S Vallarino JG. Sugar signaling during fruit ripening. Front Plant Sci. (2020) 11:564917. 10.3389/fpls.2020.564917 32983216 PMC7485278

[33] Beauvoit B Belouah I Bertin N Cakpo CB Colombié S Dai Z. Putting primary metabolism into perspective to obtain better fruits. Ann Bot. (2018) 122:1–21. 10.1093/aob/mcy057 29718072 PMC6025238

[34] Etienne A Génard M Lobit P. Mbeguié-A-Mbéguié D, Bugaud C. What controls fleshy fruit acidity? A review of malate and citrate accumulation in fruit cells. J Exp Bot. (2013) 64:1451–69. 10.1093/jxb/ert035 23408829

[35] Batista-Silva W Nascimento VL Medeiros DB Nunes-Nesi A Ribeiro DM Zsögön A. Modifications in organic acid profiles during fruit development and ripening: correlation or causation? Front Plant Sci. (2018) 9:1689. 10.3389/fpls.2018.01689 30524461 PMC6256983

[36] Pires TC Dias MI Barros L Alves MJ Oliveira MBP Santos-Buelga C. Antioxidant and antimicrobial properties of dried Portuguese apple variety (*Malus domestica* Borkh. cv Bravo de Esmolfe*). Food Chem*. (2018) 240:701–6. 10.1016/j.foodchem.2017.08.010 28946332

[37] Ehling S Cole S. Analysis of organic acids in fruit juices by liquid chromatography–mass spectrometry: an enhanced tool for authenticity testing. J Agric Food Chem. (2011) 59:2229–34. 10.1021/jf104527e 21361392

[38] Wicklund T Guyot S Le Quéré J-M. Chemical composition of apples cultivated in Norway. Crops. (2021) 1:8–19. 10.3390/crops1010003

[39] Ma BQ Liao L Zheng HY Chen J Wu BH Ogutu C. Genes encoding aluminum-activated malate transporter II and their association with fruit acidity in apple. Plant Genome. (2015) 8:1–14. 10.3835/plantgenome2015.03.0016 33228269

[40] Fallahi E Fallahi B Neilsen GH Neilsen D Peryea FJ. Effects of mineral nutrition on fruit quality and nutritional disorders in apples. Acta Hortic. (2010) 868:49–60. 10.17660/ActaHortic.2010.868.3

[41] Łysiak GP Michalska-Ciechanowska A Wojdyło A. Postharvest changes in phenolic compounds and antioxidant capacity of apples cv. Jonagold growing in different locations in Europe. Food Chem. (2020) 310:125912. 10.1016/j.foodchem.2019.125912 31841938

[42] Malec M Le Quéré JM Sotin H Kolodziejczyk K Bauduin R Guyot S. Polyphenol profiling of a red-fleshed apple cultivar and evaluation of the color extractability and stability in the juice. J Agric Food Chem. (2014) 62:6944–54. 10.1021/jf500336v 24655330

[43] Suárez B Álvarez Á L García YD. del Barrio G, Lobo AP, Parra F. Phenolic profiles, antioxidant activity and *in vitro* antiviral properties of apple pomace. Food Chem. (2010) 120:339–42. 10.1016/j.foodchem.2009.09.073

[44] Lee J Chan BLS Mitchell AE. Identification/quantification of free and bound phenolic acids in peel and pulp ofb apples (*Malus domestica*) using high resolution mass spectrometry (HRMS). Food Chem. (2017) 215:301–10. 10.1016/j.foodchem.2016.07.166 27542479

[45] He W Laaksonen O Tian Y Heinonen M Bitz L. Yang B. Phenolic compound profiles in Finnish apple (Malus × domestica Borkh) juices and ciders fermented with Saccharomyces cerevisiae and Schizosaccharomyces pombe strains. Food Chem. (2022) 373:131437. 10.1016/j.foodchem.2021.131437 34749087

[46] Mari A Tedesco I Nappo A Russo GL Malorni A Carbone V. Phenolic compound characterisation and antiproliferative activity of “Annurca” apple, a southern Italian cultivar. Food Chem. (2010) 123:157–64. 10.1016/j.foodchem.2010.04.023

[47] da Silva LC Souza MC Sumere BR Silva LG da Cunha DT Barbero GF. Simultaneous extraction and separation of bioactive compounds from apple pomace using pressurized liquids coupled on-line with solid-phase extraction. Food Chem. (2020) 318:126450. 10.1016/j.foodchem.2020.126450 32151921

[48] Sánchez-Rabaneda F Jáuregui O Lamuela-Raventós RM Viladomat F Bastida J Codina C. Qualitative analysis of phenolic compounds in apple pomace using liquid chromatography coupled to mass spectrometry in tandem mode. Rapid Commun Mass Spectrom. (2004) 18:553–63. 10.1002/rcm.1370 14978800

[49] Hu Q Chen YY Jiao QY Khan A Shan J Cao GD Li F. Polyphenolic compounds from Malus hupehensis and their free radical scavenging effects. Nat Prod Res. (2017) 32:2152–8. 10.1080/14786419.2017.1367784 28901161

[50] López V Les F Mevi S Nkuimi Wandjou JG Cásedas G Caprioli G. Phytochemicals and enzyme inhibitory capacities of the methanolic extracts from the Italian apple cultivar Mela Rosa dei Monti Sibillini. Pharmaceuticals. (2020) 13:127. 10.3390/ph13060127 32580356 PMC7344947

[51] Fotirić Akšić M Dabić Zagorac D Sredojević M Milivojević J Gašić U Meland M. Chemometric characterization of strawberries and blueberries according to their phenolic profile: Combined effect of cultivar and cultivation system. Molecules. (2019) 24:4310. 10.3390/molecules24234310 31779117 PMC6930459

[52] Tan L Jin Z Ge Y Nadeem H Cheng Z Azeem F Zhan R. Comprehensive ESI-Q TRAP-MS/MS based characterization of metabolome of two mango (*Mangifera indica* L) cultivars from China. Sci Rep. (2020) 10:1–19. 10.1038/s41598-020-75636-y 33208758 PMC7676270

[53] Guo J Yue T Yuan Y Wang Y. Chemometric classification of apple juices according to variety and geographical origin based on polyphenolic profiles. J Agric Food Chem. (2013) 61:6949–63. 10.1021/jf4011774 23815505

[54] Ouyang H Li J Wu B Zhang X Li Y Yang S. robust platform based on ultra-high performance liquid chromatography Quadrupole time of flight tandem mass spectrometry with a two-step data mining strategy in the investigation, classification, and identification of chlorogenic acids in *Ainsliaea fragrans* Champ. J Chromatogr A. (2017) 1502:38–50. 10.1016/j.chroma.2017.04.051 28477946

[55] Ochman H Lawrence JG Groisman EA. Lateral gene transfer and the nature of bacterial innovation. Nature. (2020) 405:299–304. 10.1038/35012500 10830951

[56] Park EK Ahn SR Kim DH Lee EW Kwon HJ Kim BW. Effects of unripe apple polyphenols on the expression of matrix metalloproteinase-1 and type-1 procollagen in ultraviolet irradiated human skin fibroblasts. J Korean Soc Appl Biol Chem. (2014) 57:449–55. 10.1007/s13765-014-4128-7

[57] Jaiswal R Karar MGE Gadir HA. Kuhnert N. Identification and characterisation of phenolics from *Ixora coccinea* L (Rubiaceae) by liquid chromatography multi-stage mass spectrometry. Phytochem Anal. (2014) 25:567–76. 10.1002/pca.2530 25185927

[58] Liu M Huang X Liu Q Chen M Liao S Zhu F. Rapid screening and identification of antioxidants in the leaves of *Malus hupehensis* using off-line two-dimensional HPLC–UV–MS/MS coupled with a 1, 1′-diphenyl-2-picrylhydrazyl assay. J Sep Sci. (2018) 41:2536–43. 10.1002/jssc.201800007 29667362

[59] De Paepe D Servaes K Noten B Diels L De Loose M Van Droogenbroeck B. An improved mass spectrometric method for identification and quantification of phenolic compounds in apple fruits. Food Chem. (2013) 136:368–75. 10.1016/j.foodchem.2012.08.062 23122072

[60] Kumar S Pandey KA. Chemistry and biological activities of flavonoids: an overview. Sci World J. (2013) 2013:162750. 10.1155/2013/162750 24470791 PMC3891543

[61] Demirci MA Ipek Y Gul F Ozen T Demirtas I. Extraction, isolation of heat-resistance phenolic compounds, antioxidant properties, characterization and purification of 5-hydroxymaltol from Turkish apple pulps. Food Chem. (2018) 269:111–7. 10.1016/j.foodchem.2018.06.147 30100412

[62] Fazio G Cheng L Grusak MA Robinson TL. Apple rootstocks influence mineral nutrient concentration of leaves and fruit. New York Fruit Quarterly. (2015) 25:11–5.

[63] Fazio G Lordan J Grusak MA Francescatto P Robinson TLI. Mineral nutrient profiles and relationships of ‘Honeycrisp' grown on a genetically diverse set of rootstocks under Western New York climatic conditions. Sci Hortic. (2020) 266:108477. 10.1016/j.scienta.2019.05.004

