# Supplementary material for: Polyphenolics and Chemical Profiles of Domestic Norwegian Apple (Malus × domestica Borkh.) Cultivars
Source: Front Nutr. 2022 Jun 30;9:941487. doi: 10.3389/fnut.2022.941487 (PMC9280294; doi:10.3389/fnut.2022.941487)
Supplement: Supplementary file 1 [file Data_Sheet_1.DOCX]

***Supplementary Material***

# 1 Supplementary Tables Legend:

**Supplementary Table S1.** Apple varieties from two sampling sites in Norway, Norwegian Institute of Bioeconomy Research, the Ullensvang area (NIBIO) and Njøs Fruit and Berry Centre.

**Supplementary Table S2.** Instrumental operating conditions for ICP-OES.

**Supplementary Table S3.** Experimental conditions of mobile phase for sugar and sugar alcohol determination by IC.

# 2 Supplementary Figures Legend:

**Supplementary Figure S1**. PCA ordination of sugars content variables: **(A)** projection in PC1-PC2 plane; **(B)** projection in PC1-PC3 plane.

**Supplementary Figure S2.** PCA ordination of fruit acids content variables: **(A)** projection in PC1-PC2 plane; **(B)** projection in PC1-PC3 plane.

**Supplementary Figure S3.** PCA ordination of element content variables: **(A)** projection in PC1-PC2 plane; **(B)** projection in PC1-PC3 plane.

**Supplementary Figure S4.** PCA ordination of phenolic content, TPC, and RSA variables: **(A)** projection in PC1-PC2 plane; **(B)** projection in PC1-PC3 plane.

.**Supplementary Table S1.** Apple varieties from two sampling sites in Norway, Norwegian Institute of Bioeconomy Research, the Ullensvang area (NIBIO) and Njøs Fruit and Berry Centre.

| **NIBIO area** | | | |  | **Njøs** | |
| --- | --- | --- | --- | --- | --- | --- |
| **Sample No.** | **Apple variety** | **Sample No.** | **Apple variety** |  | **Sample No.** | **Apple variety** |
| **1** | Åkerø | **38** | Maglemer |  | **75** | Aagoteple |
| **2** | Aroma Ylvisåker | **39** | Nanna |  | **76** | Antonovka |
| **3** | Astrakan | **40** | Ølands kungseple |  | **77** | Apalseteple |
| **4** | Beauty of Bath | **41** | Øskaug |  | **78** | Bøtuneple |
| **5** | Brureple | **42** | Paradiseple |  | **79** | Edholm |
| **6** | Charlamowsky | **43** | Petter Heyerdal |  | **80** | Ekely |
| **7** | Cox Pomona | **44** | Prins |  | **81** | Enestaende |
| **8** | Discovery | **45** | Quinte |  | **82** | Filippa |
| **9** | Early Red Bird | **46** | Raud Torstein |  | **83** | Franskar |
| **10** | Eir | **47** | Raud Gravenstein |  | **84** | Fuhr |
| **11** | Elstar | **48** | Raud Sävstaholm |  | **85** | Furuholm |
| **12** | Fosseple | **49** | Raud Sommerkavill |  | **86** | Gloppestadeple |
| **13** | Franskar | **50** | Raudt Laupsaeple |  | **87** | Grågylling |
| **14** | Fristeren | **51** | Rondestveit |  | **88** | Grindeeple |
| **15** | Fuhr | **52** | Rossvolleple |  | **89** | Jordbæreple |
| **16** | Furuholm | **53** | Rubin |  | **90** | Kjerringholm |
| **17** | Garborg | **54** | Sävstaholm |  | **91** | Lærdalseple |
| **18** | Geneva Early | **55** | Silkeeple |  | **92** | Laveple |
| **19** | Gravenstein | **56** | Sitroneple |  | **93** | Lavoll |
| **20** | Grønt Laupsaeple | **57** | Siv |  | **94** | Leriseple |
| **21** | Gul Granat | **58** | Stor Granat |  | **95** | Løeple |
| **22** | Gul richard | **59** | Storesteinseple |  | **96** | Nanseneple |
| **23** | Gullspir | **60** | Summered |  | **97** | Ørekrok |
| **24** | Gyldenkroks Astrakan | **61** | Sunrise |  | **98** | Øysteineple |
| **25** | Haugeeple | **62** | Sylvia |  | **99** | Petrineeple |
| **26** | Haugmann | **63** | Sysekavil |  | **100** | Skredsvigeple |
| **27** | Hjartneseple | **64** | Tohoku 2 |  | **101** | Stor Torstein |
| **28** | Julyred | **65** | Tokheimseple |  | **102** | Sukkereple |
| **29** | Katja | **66** | Tormodseple |  | **103** | Vågaeple |
| **30** | Kaupanger | **67** | Transparente Blanche |  |  |  |
| **31** | Kavil | **68** | Tveiteple |  |  |  |
| **32** | Kviteple | **69** | Ulgenes |  |  |  |
| **33** | Langballeeple | **70** | Ullerneple |  |  |  |
| **34** | Leiknes | **71** | Vanleg Torstein |  |  |  |
| **35** | Leinestrand | **72** | Vinterrosenstrips |  |  |  |
| **36** | Lobo | **73** | Vista Bella |  |  |  |
| **37** | Løeple | **74** | Worcester Permain |  |  |  |

**Supplementary Table S2.** Instrumental operating conditions for ICP-OES.

| **Parameter** | **Value** |
| --- | --- |
| Radio frequency power (RF) | 1150 W |
| Plasma view | Axial |
| Nebulizer | Standard glass concentric |
| Spray chamber | Standard glass cyclonic |
| Pump tubing (Tygon) | Sample (Orange-White)  Drain (White-White) |
| Ceramic centre tube | 2 mm |
| Purge gas | Argon |
| Nebulizer argon flow | 0.50 L/min |
| Auxiliary  argon  flow | 0.50 L/min |
| Coolant  argon  flow | 12 L/min |
| Sample flush time | 40 s |
| Analysis pump rate | 50 rpm |
| Integration times     Low (166 - 230 nm)                                  High (230 - 847 nm) | 15 s  5 s |
| Analysis mode | Speed |
| Software | iTEVA |

**Supplementary Table S3.** Experimental conditions of mobile phase for sugars and sugar alcohols determination by IC.

| **Time** | **Water** | **600 mM sodium hydroxide** | **500 mM sodium acetate trihidrate** |
| --- | --- | --- | --- |
| Equilibration 30 min | 85 | 15 | 0 |
| 0.0 – 5.0 | 85 | 15 | 0 |
| 5.0 - 5.1 | 83 | 15 | 2 |
| 5.1 - 12.0 | 83 | 15 | 2 |
| 12.0 - 12.1 | 81 | 15 | 4 |
| 12.1 - 20.0 | 81 | 15 | 4 |
| 20.0 - 20.1 | 60 | 20 | 20 |
| 20.1 - 30.0 | 60 | 20 | 20 |
