# Supplementary figures and images for: Polyphenolics and Chemical Profiles of Domestic Norwegian Apple (Malus × domestica Borkh.) Cultivars
Source: Front Nutr. 2022 Jun 30;9:941487. doi: 10.3389/fnut.2022.941487 (PMC9280294; doi:10.3389/fnut.2022.941487)

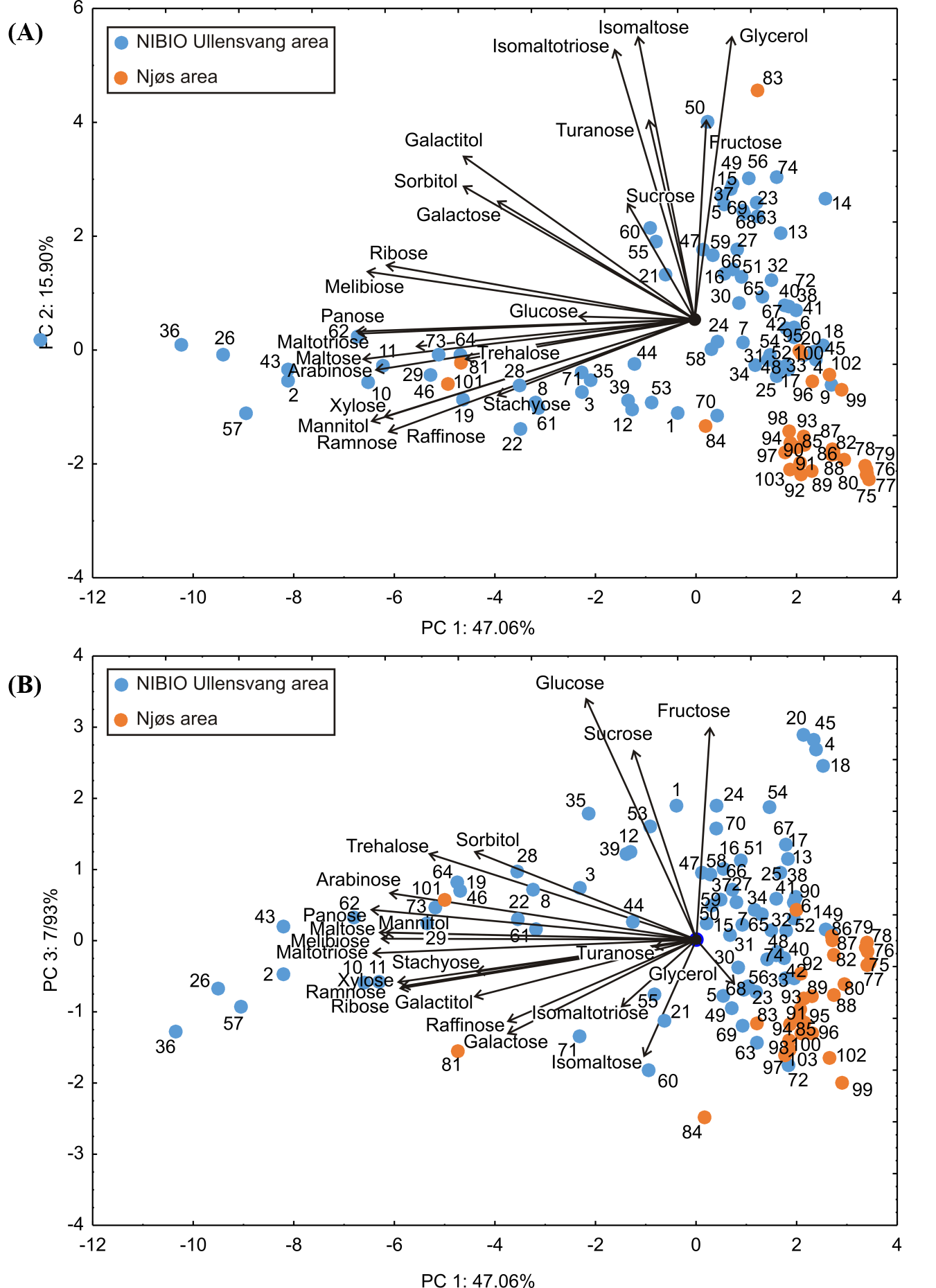

Supplement: Supplementary file 2 [file Image_1.TIFF]

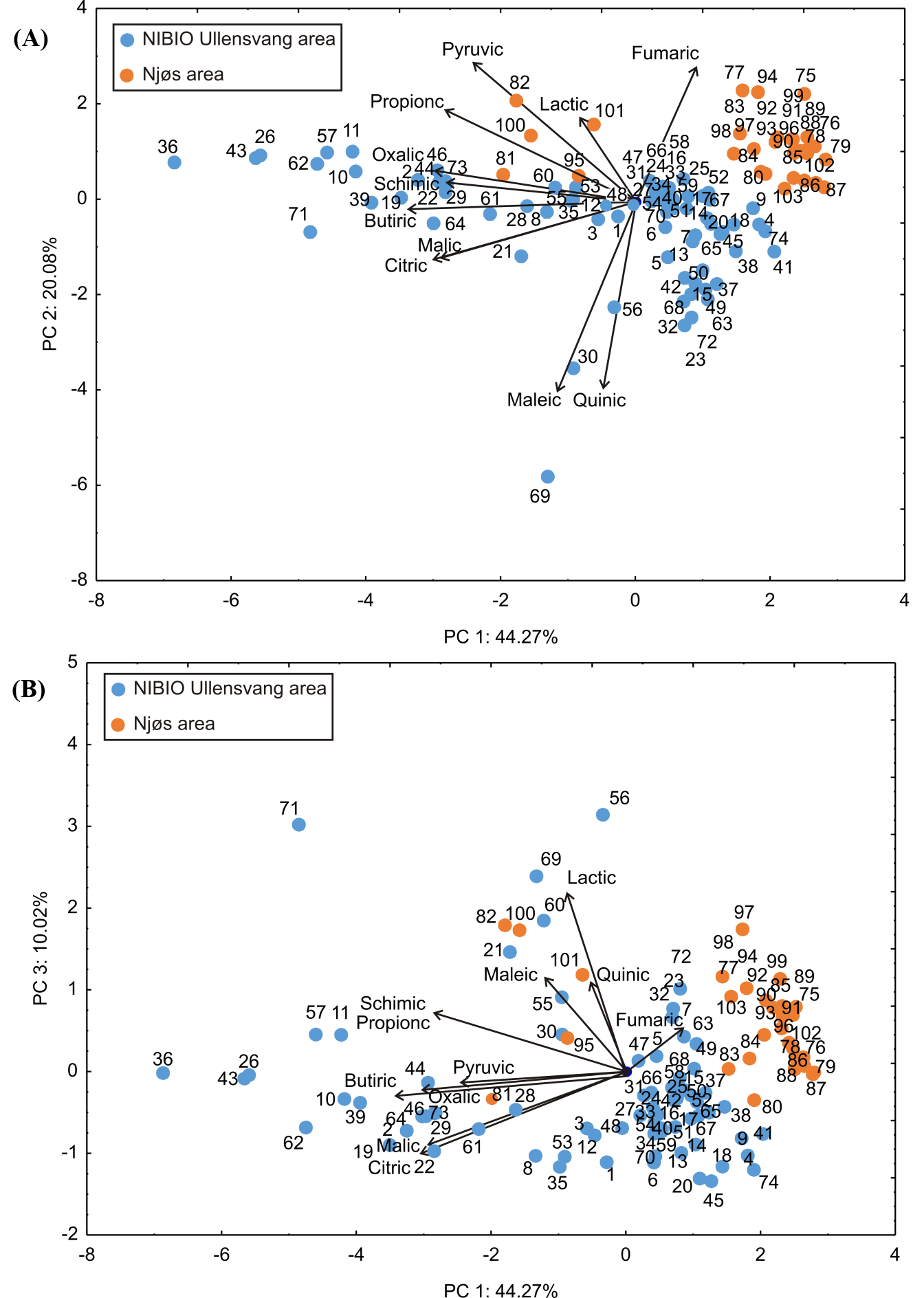

Supplement: Supplementary file 3 [file Image_2.TIFF]

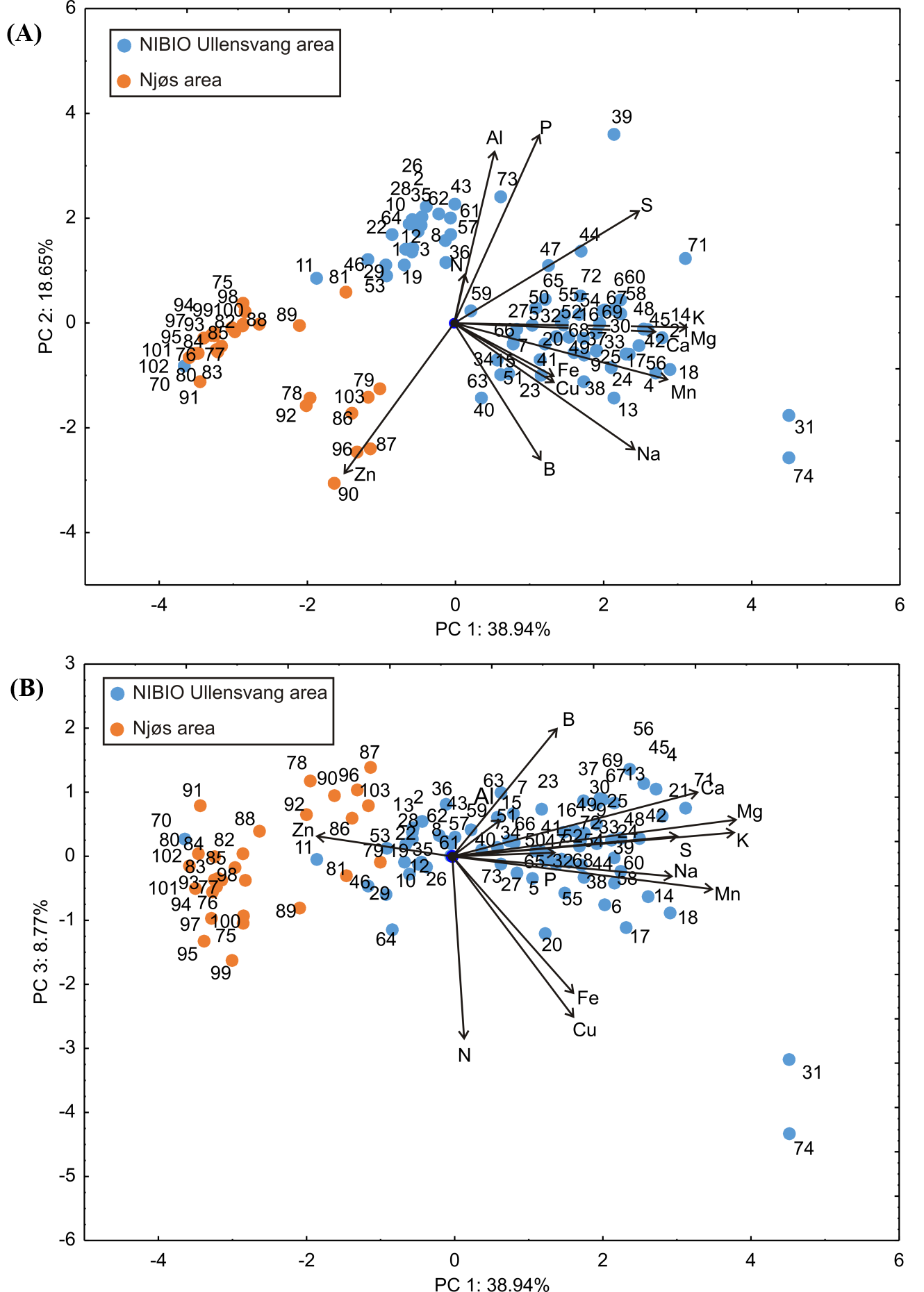

Supplement: Supplementary file 4 [file Image_3.TIFF]

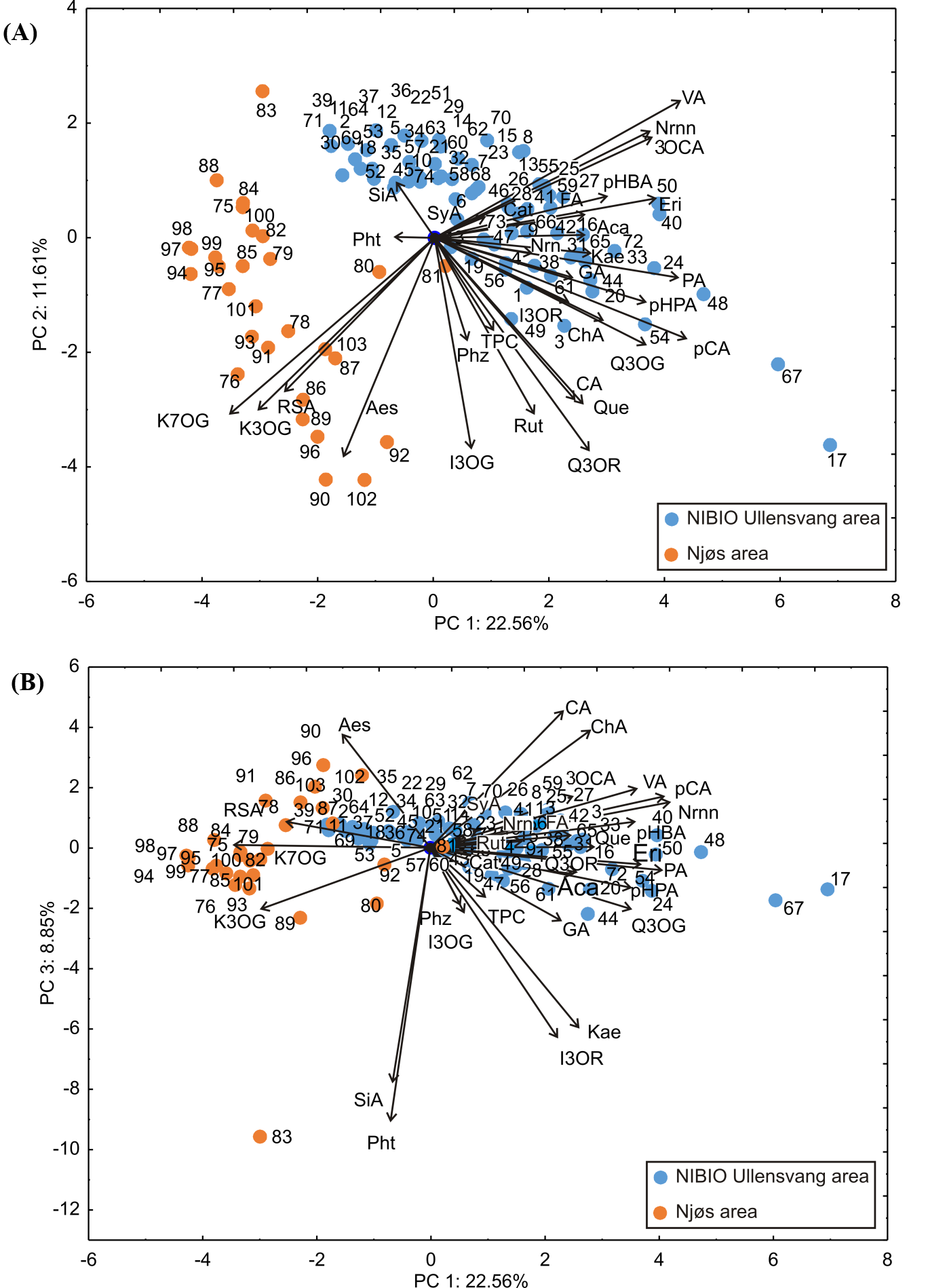

Supplement: Supplementary file 5 [file Image_4.TIFF]
